# Metabolomic profiling of diatoms reveals distinct impacts of silver nanoparticles and ions

**DOI:** 10.1039/d6en00326e

**Published:** 2026-06-04

**Authors:** Arin Kantarciyan, Inés Segovia-Campos, Matea Marelja, Rocco Gasco, Weiwei Li, Arturo A. Keller, Vera I. Slaveykova

**Affiliations:** a University of Geneva, Faculty of Sciences, Department F.-A. Forel for Environmental and Aquatic Sciences, Environmental Biogeochemistry and Ecotoxicology Bvd Carl-Vogt 66 1205 Geneva Switzerland vera.slaveykova@unige.ch +41 22 379 0335; b Bren School of Environmental Science & Management, University of California Santa Barbara CA 93106-5131 USA

## Abstract

Silver nanoparticles (nAg) raise ecotoxicological concerns due to their increasing production, application, and inevitable release into aquatic environments. In freshwater, nAg aggregate and partially dissolve, coexisting with dissolved silver (Ag^+^). Diatoms, key phytoplankton contributing to global silicon and carbon cycles, are particularly relevant for assessing silver toxicity, yet the metabolic impacts of Ag^+^ and nAg remain poorly understood. Here, we combined metabolomics and physiological assays to investigate the effects of short-term (2 h), sublethal exposures of Ag^+^ (0.01 and 0.02 mg L^−1^) and nAg (0.1 and 0.3 mg L^−1^) on the freshwater diatom *Cyclotella meneghiniana*. Both silver forms strongly affected the diatom metabolism, with notable alterations in amino acid, polyamine, glutathione, and nucleotide metabolism, as well as the tricarboxylic acid (TCA) cycle. These metabolic shifts were dependent on the silver form and concentration, and correlated with intracellular silver accumulation. Notably, nAg exerted stronger and more specific effects on the TCA cycle, glutathione metabolism, and polyamine synthesis than Ag^+^. Physiologically, both nAg and Ag^+^ impaired photosystem performance, but only nAg induced enhanced reactive oxygen species (ROS) production and altered carbonic anhydrase activity. This study provides the first insights into both shared and distinct metabolomic responses of diatoms to short-term exposure to Ag^+^ and nAg, highlighting the greater specificity and potency of nAg in disrupting key metabolic and physiological processes.

## Abstract 2

Sub-lethal exposure to dissolved and nanoparticulate silver induces distinct and substantial alterations in diatom metabolism, with central carbon and nitrogen metabolic pathways exhibiting particularly pronounced perturbations.

## Graphical abstract

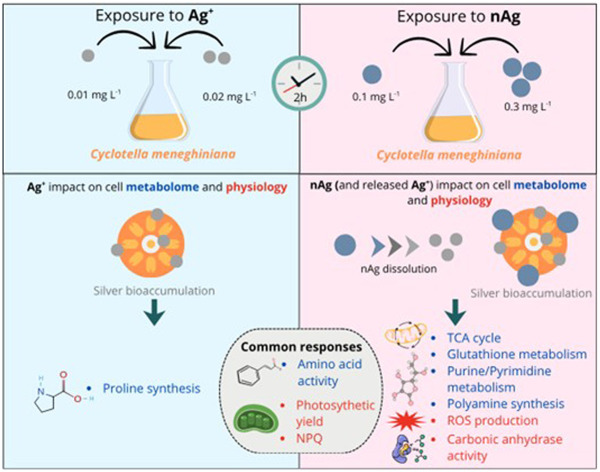

**Environmental significance**

Silver nanoparticles (nAg) are raising significant ecotoxicological concerns due to their increasing production, widespread applications, and inevitable release into the environment. Diatoms are primary producers and key drivers of biogeochemical cycles; however, their metabolic responses to nAg remain poorly understood. This study demonstrates that sub-lethal concentrations of both dissolved and nanoparticulate silver distinctly and profoundly affect diatom metabolism, with central carbon and nitrogen pathways particularly disrupted. The findings further reveal that nAg exert stronger and differential impacts compared to dissolved silver. Such impairments in diatom metabolic functioning could compromise their ecological role, ultimately affecting nutrient cycling, food web dynamics, and ecosystem stability in freshwater environments exposed to nAg.

## 1. Introduction

Engineered nanomaterials (ENMs) have raised increasing environmental concerns due to their widespread applications and enhanced physicochemical properties.^1,2^ Among these, silver nanoparticles (nAg) are particularly prominent owing to their broad-spectrum antimicrobial activity, including antibacterial, antifungal, and antiviral effects.^3,4^ As their production, application, and disposal continue to rise, nAg inevitably enter aquatic environments, where they interact with a variety of organisms, including phytoplankton.^5,6^ The persistence of nAg in water depends on environmental conditions and physicochemical properties, undergoing transformations, such as aggregation and release ionic silver (Ag^+^).^2,7,8^ As a result, the observed toxic effects on phytoplankton are typically attributed to a combined action of both nAg and Ag^+^.^9^ The impacts of nAg and Ag^+^ on phytoplankton physiology are well documented, with numerous studies reporting growth inhibition, photosystem impairment, enhanced reactive oxygen species (ROS) generation, and disruption of membrane integrity.^5,10^ However, such physiological endpoints often miss subtle, early-stage cellular perturbations and may not fully reveal the differential effects exerted by the two distinct forms of silver.

To capture these early and often overlooked responses, metabolomics provides a powerful approach, enabling the comprehensive profiling of low-molecular-weight metabolites that reflect the organism's biochemical activity and, consequently, its physiological state. Indeed, several studies have employed metabolomics to investigate the impact of nAg and Ag^+^ on phytoplankton species. For instance, in *Chlorella vulgaris*, nAg altered phosphorous and glutathione metabolism^11^ and induced particle size-dependent metabolic toxicity, affecting pathways such glycerophospholipid metabolism and protein biosynthesis.^12^ In another green alga, *Chlorella pyrenoidosa*, metabolic responses to nAg varied with exposure conditions: single exposures induced stronger toxicity than repeated exposures and primarily disrupted central nitrogen and carbon metabolism,^13^ while co-exposure with hematite nanoparticles further amplified metabolic perturbations compared with nAg alone.^14^ Although these studies addressed diverse aspects of nAg toxicity, limited experimental data on Ag^+^ effects have made it difficult to disentangle the specific contributions of nAg to the observed metabolic responses.

Nevertheless, some studies have highlighted the role of Ag^+^ in the overall toxicity of nAg. In cyanobacterium *Microcystis aeruginosa*, most metabolic responses to nAg and Ag^+^ were shared, although certain pathways, such as arginine and proline metabolism, indole biosynthesis, and phospholipid metabolism, were regulated exclusively by nAg.^15^ In contrast, in cyanobacterium *Nostoc sphaeroides*, Ag^+^ exerted a stronger negative effect on antioxidant defenses than nAg, with associated metabolites decreasing exclusively under Ag^+^ exposure and a more extensive metabolic reprogramming.^16^ Similarly, in the mixotrophic flagellate *Poterioochromonas malhamensis*, no nAg-specific metabolic perturbations were detected, although nAg contributed to alterations in amino acid metabolism, the tricarboxylic acid (TCA) cycle, and oxidative stress responses during the early exposure.^17^ Collectively, these studies on both silver forms provide valuable insights but yield species-specific metabolic outcomes, highlighting the need for broader investigations across diverse phytoplankton taxa.

Diatoms, ubiquitous in aquatic ecosystems, account for ∼20–25% of global primary production and are central to silicon and carbon cycles. Their need for dissolved silica and uptake of inorganic carbon during photosynthesis and growth links these biogeochemical processes and drives CO_2_ sequestration.^18,19^ Due to their ubiquity, adaptability, and sensitivity to environmental change, diatoms are widely used as ecological indicators, particularly for early detection of water quality shifts.^20,21^ Despite their ecological significance and their established role in biomonitoring, the metabolic responses of diatoms to both nAg and Ag^+^ remain largely unexplored.

In this study, we investigate the short-term physiological and metabolic responses of the ecologically relevant diatom *Cyclotella meneghiniana*, following exposure to both Ag^+^ and nAg. Its silica frustule, characterized by nanometric porosity, represents a critical nano-bio interface that may modulate nanoparticle interaction, uptake and associated metabolic responses.^22^ Using a combination of targeted metabolomics, ecotoxicological and enzymatic assays we address the following key questions: (i) what key metabolic changes occur in *C. meneghiniana* when exposed to Ag^+^ and nAg? (ii) do these metabolic responses follow a concentration-dependent pattern? and (iii) how do the metabolic effects differ between Ag^+^ and nAg, and where do they overlap?

## 2. Materials and methods

### 2.1. nAg characterization

Citrate coated nAg with a primary size of 20 nm (1 g mL^−1^ in 2 mmol L^−1^ citrate) were purchased from NanoComposix (San Diego, CA, USA). Ionic silver standard solution (1 g L^−1^ Ag^+^ in 2% HNO_3_, TraceCERT) was obtained from Sigma-Aldrich (Buchs, Switzerland). Nanoparticles in citrate solution had a diameter of 19.9 ± 2.8 nm, a hydrodynamic diameter of 32.93 ± 0.14 nm, a *z*-potential of −39.93 ± 1.62 mV, and a polydispersity index (PDI) of 0.265 ± 0.001. The characteristics of nAg suspension in synthetic freshwater medium (SFM), which served as the exposure medium, were studied following a previously developed approach.^23^ UV-vis absorbance spectra of nAg, exhibiting a characteristic surface plasmon resonance (SPR) peak at ∼390 nm, were recorded 2 min and 2 h after resuspension in SFM using BioTek Synergy H1 Hybrid (Bucher Biotec, Switzerland). The hydrodynamic size distribution and *z*-potential were measured by Zetasizer Nano ZS (Malvern, UK), and nanoparticle aggregation was further characterized by transmission electron microscopy (TEM, Talos™ L120C TEM, Thermo Fisher, USA). The dissolution of nAg was quantified under cell-free conditions using a centrifugation-based method with minor modifications.^23^ Briefly, the nAg suspensions were centrifuged for 3 h (24 000 × *g* and 4 °C) and the supernatant was collected and acidified to 2% HNO_3_ (v : v) before being analyzed with inductively coupled plasma mass spectrometry (ICP-MS; 7700x, Agilent, USA).

### 2.2. Diatom culture and exposure to nAg and Ag^+^

The diatom *C. meneghiniana* (CCAC 0039) was obtained from the Central Collection of Algal Cultures (CCAC) at the University of Duisburg-Essen, Germany. The cells were axenically cultured in SFM medium containing Si (Table S1). Cultures were grown in a controlled chamber (Binder KBW 720, Binder GmBH, Tuttlingen, Germany) at 20 °C under a light intensity of 90 μmol s^−1^ m^−2^ with a 16 : 8 light and dark cycle. Cells were maintained on continuous orbital shaking of 60 rpm. The cell density was monitored *via* flow cytometry (FCM) (BD Accuri C6, BD Biosciences, San Jose, CA, USA). Diatom cells in the mid-exponential growth phase were harvested and rinsed with fresh SFM exposure medium (Table S1). The cells were then resuspended to reach a final cell density of 1 × 10^6^ cells per mL in SFM exposure medium containing nAg or Ag^+^.

**Fig. 1:**
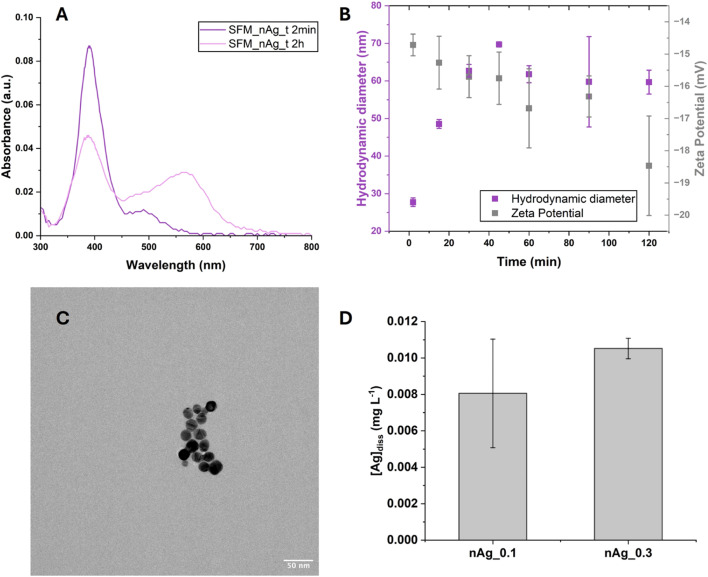
Characteristics of nAg suspensions in SFM exposure medium over 2 h. (A) SPR-UV-vis absorbance spectra. (B) Hydrodynamic diameter and *z*-potential of 1 mg L^−1^ nAg in SFM up to 2 h. (C) TEM image of nAg in SFM after 2 h. (D) Dissolved Ag concentrations (mg L^−1^) in two nAg suspensions (nAg_0.1: 0.1 mg L^−1^; nAg_0.3: 0.3 mg L^−1^) in SFM after 2 h. Error bars represent standard deviation (*n* = 3).

Five conditions were tested, including unexposed cells that were used as control (CTR), cells exposed to 0.01 and 0.02 mg L^−1^ Ag^+^ and 0.1 and 0.3 mg L^−1^ nAg. Tested concentrations corresponded to the EC_20_ and EC_50_ values at 72 h for *C. meneghiniana* when exposed to both Ag^+^ and nAg (Fig. S1). A short exposure period of 2 h was chosen to investigate the early effects of nAg and Ag^+^ on diatom metabolism without compromising the growth. Additionally, the concentration of 0.01 mg L^−1^ Ag^+^ was selected as it corresponded to dissolved silver content in 0.3 mg L^−1^ nAg suspension after 2 h in SFM exposure medium (Fig. 1), allowing for a direct comparison between the effects of nAg and Ag^+^. Finally, intracellular and adsorbed silver fractions by *C. meneghiniana* were quantified after 2 h exposure to both Ag^+^ and nAg a following previously optimized procedure.^22^ Experimental details can be found in the SI.

### 2.3. Physiological endpoint assessment

Several physiological endpoints were assessed in cultures of the diatom *C. meneghiniana* (∼6 × 10^5^ cells per mL) following 2 h exposure to silver, either as Ag^+^ (0.01 and 0.02 mg L^−1^) or nAg (0.1 and 0.3 mg L^−1^). Following exposure, cell aliquots were collected to assess photosystem performance and membrane integrity. ***Photosynthetic activity*** was evaluated using a FluorCam 800MF (Drásov, Czech Republic) in pulse amplitude modulated (PAM) mode, measuring the maximum quantum yield (Fv/Fm) and non-photochemical quenching (NPQ). ***Membrane permeability*** of Ag-exposed diatom cells was estimated *via* propidium iodide (PI) based flow cytometry assay, following the method described in ref. 22. The remaining culture was homogenized by sonication to assess total intracellular ROS, carbonic anhydrase (CA) and glutathione peroxidase (GSH-Px) activity. ***Total ROS levels*** were quantified using the fluorescent probe 5-(and-6)-chloromethyl-2′,7′-dichlorodihydrofluorescein diacetate (CM-H_2_DCFDA) (MedChemExpress LLC, Monmouth Junction, USA).^24^***CA activity*** was determined using the electrometric Wilbur–Anderson method based on pH variation, with minor modifications.^25^ Finally, ***GSH-Px activity*** was determined following instructions provided by the activity kit (Merck KGaA, Darmstadt, Germany). Data of physiological responses were analyzed using one-way analysis of variance (ANOVA) followed by Tukey's *post hoc* test integrated in OriginPro 2024 software. Detailed experimental parameters and protocols are provided in the SI.

### 2.4. Liquid chromatography-mass spectrometry (LC-MS) targeted metabolomics

After 2 h exposure to silver, 400 mL of cell aliquots were harvested and rinsed with fresh, silver-free, SFM exposure medium to eliminate extracellular metabolites which could interfere with the measurements. Then, harvested cell pellets were flash frozen in liquid nitrogen to stop metabolic activity and freeze-dried (Beta 1–8 LSCplus, Christ®, Martin Christ Gefriertrocknungsanlage GmbH, Germany). Metabolites, including amino acids (AA), antioxidants (AO), organic acids (OA)/phenolics, nucleobases/sides/tides, sugars/sugar alcohols, polyamines (PA), pigments and fatty acids (FA), were extracted using 80% methanol containing 2% formic acid following a previously established protocol.^26,27^ Targeted metabolomic analysis was performed using an Agilent 6470 liquid chromatography triple quadrupole mass spectrometer with MS parameters provided in SI (Table S2). Statistical analysis of the targeted metabolomics data was performed using MetaboAnalyst 6.0,^28^ following previously established approaches.^17,29^ Comprehensive details of data processing, statistical analysis, and pathway analysis can be found in the SI.

## 3. Results and discussion

### 3.1. nAg characterization in exposure medium

nAg suspensions in SFM exhibited a characteristic absorbance peak at 391 nm (Fig. 1A). Over a 2 h period, the intensity of this peak decreased, and a secondary peak emerged at higher wavelengths (Fig. 1A), indicating aggregation.^23^ In agreement with these observations, the hydrodynamic diameter of nAg increased from 27.73 ± 1.15 nm to 59.70 ± 3.17 nm, and the presence of aggregates was confirmed by TEM (Fig. 1B and C). nAg stability not only depends on the surface coating but also on the ionic strength of the medium where they are resuspended. It is well documented that the moderate ionic strength of SFM (6.65 mM) promotes aggregation,^30^ potentially limiting nano-bio interactions through a reduced surface area-to-volume ratio^31^ and consequently reducing nAg bioavailability to *C. meneghiniana*.

Immediately after resuspension of nAg in SFM exposure medium, the absolute *z*-potential values decreased, reaching −18.47 ± 1.55 mV after 2 h (compared to −39.9 ± 1.62 mV in citrate solution) (Fig. 1B), indicating a partial surface charge screening due to the presence of mono and divalent cations in the SFM medium.^23^ Dissolved Ag^+^ concentrations measured in cell-free exposure medium after 2 h were low for both nAg suspensions, reaching 0.008 ± 0.003 mg L^−1^ and 0.011 ± 0.001 mg L^−1^ Ag for nAg_0.1 and nAg_0.3, respectively (Fig. 1D). Given that nAg toxicity is frequently attributed to Ag^+^,^32^ the low dissolution observed in this study may indicate a limited contribution of the ionic form to toxicity toward *C. meneghiniana*.

### 3.2. Intracellular and adsorbed cellular Ag

**Fig. 2:**
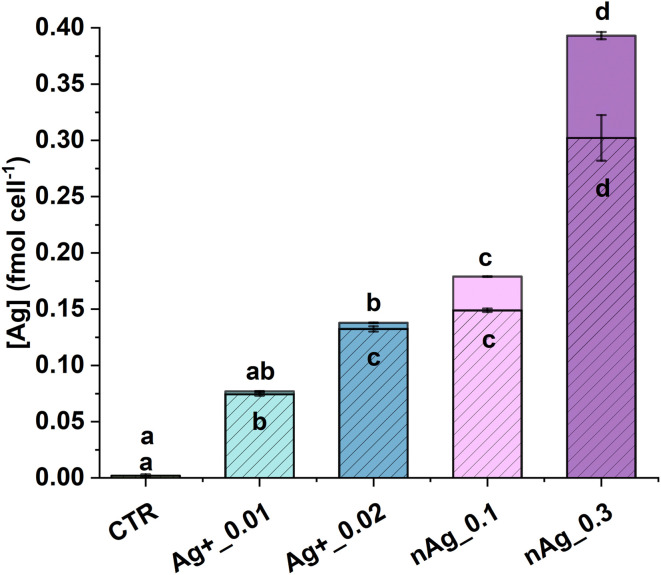
Intracellular and surface-adsorbed silver content in *C. meneghiniana* after 2 h exposure to dissolved silver (Ag^+^; 0.01 and 0.02 mg L^−1^) and nanoparticulate silver (nAg; 0.1 and 0.3 mg L^−1^). Dashed bars represent intracellular Ag content, while solid (non-dashed) bars indicate surface-adsorbed (extractable) Ag. Error bars denote standard deviation (*n* = 3). Different letters indicate statistically significant differences between exposure conditions (one-way ANOVA followed by Tukey's *post hoc* test, *p* < 0.05).

The cellular Ag burden of the diatom *C. meneghiniana* was assessed in two operationally defined fractions: surface-adsorbed and intracellular Ag. Ag removable by chemical extractants was classified as surface-adsorbed, while non-removable Ag was considered intracellular.^22^ Intracellular Ag content significantly increased with rising Ag^+^ and nAg exposures. Despite higher total Ag in the 0.1 mg L^−1^ nAg treatment compared to 0.02 mg L^−1^ Ag^+^, intracellular Ag levels were similar (Fig. 2). In addition, the amount of adsorbed Ag increased with exposure concentration; however, no significant difference was observed between the two Ag^+^ treatments (0.01 and 0.02 mg L^−1^) (Fig. 2). Unlike the comparable intracellular Ag in 0.02 mg L^−1^ Ag^+^ and 0.1 mg L^−1^ nAg, adsorbed Ag differed significantly between these treatments (Fig. 2). Overall, adsorbed Ag remained much lower than intracellular Ag, consistent with previous studies showing that Ag predominantly accumulates intracellularly in diatoms.^22,33^

Although the dissolved fractions of nAg_0.1 and nAg_0.3 were similar to that of Ag^+^_0.01 after 2 h, in the absence of cells (Fig. 1), both intracellular and adsorbed Ag burdens were significantly higher in nAg treatments than in the dissolved counterpart (Fig. 2), suggesting a potential internalization and surface adsorption of nAg. Similar behavior has been reported in other diatoms with comparable cell wall properties. *Cylindrotheca fusiformis* and *C. closterium* internalized nAg when exposed to particles with a nominal size of 25.6 ± 13.7 nm.^34^ Similarly, nAg were detected inside the cell membrane of the diatom *Thalassiosira pseudonana* following exposure to a polydisperse nAg suspension ranging from 5 to 120 nm in size.^35^ The nanoporous architecture of the frustule likely increases nAg retention at the cell surface, enhancing nAg–membrane interactions and facilitating uptake *via* vesicle-mediated pathways such as endocytosis, a proposed major entry route for nAg in cells.^34^ Adsorption of nAg followed by internalization has been suggested as a key mechanism driving cellular physiological and metabolic responses.^36,37^

### 3.3. Effects of nAg and Ag^+^ exposure on diatom physiology

**Fig. 3:**
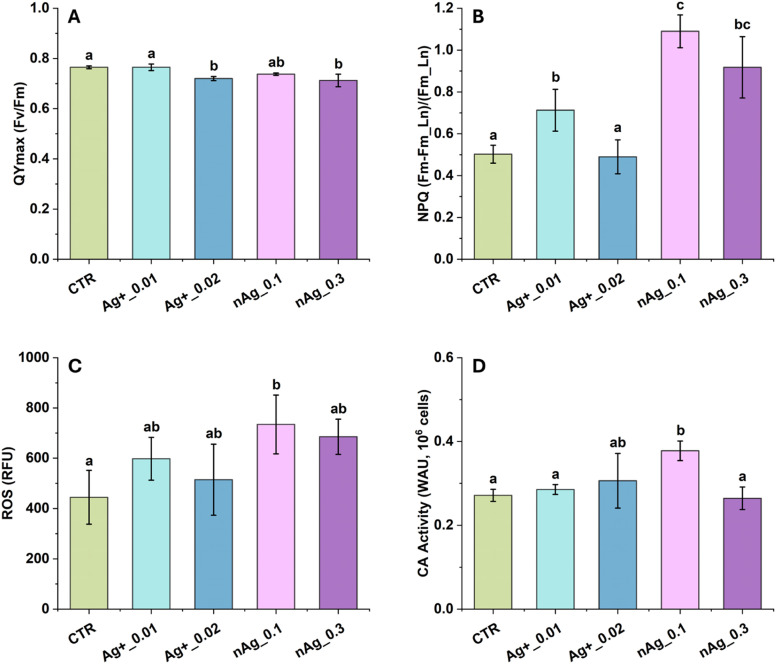
Physiological responses of diatom *C. meneghiniana* exposed to dissolved silver (Ag^+^; 0.01 and 0.02 mg L^−1^) and nanoparticulate silver (nAg; 0.1 and 0.3 mg L^−1^) for 2 h. (A) Photosynthetic yield (QYmax, Fv/Fm). (B) Non-photochemical quenching in light adapted state (NPQ, (Fm-Fm_Ln)/(Fm_Ln)). (C) Total ROS levels expressed as relative fluorescence units (RFU). (D) CA activity expressed in Wilbur–Anderson units (WAU) for 10^6^ cells. Error bars denote standard deviation (*n* = 4). Different letters indicate statistically significant differences between exposure conditions (one-way ANOVA followed by Tukey's *post hoc* test, *p* < 0.05).

Short-term exposure of the diatom *C. meneghiniana* to Ag^+^ and nAg induced multiple physiological responses (Fig. 3). Photosynthetic yield, measured as the Fv/Fm ratio, significantly decreased after 2 h of exposure to the highest Ag^+^ concentration (0.02 mg L^−1^) and to the highest nAg concentration (0.3 mg L^−1^), whereas other treatments remained comparable to the unexposed control (Fig. 3A). The Fv/Fm ratio provides insight into the functionality of photosystem II under dark-adapted conditions.^38^ Several studies have reported that nAg exposure can impair algal photosynthesis, leading to reductions in chlorophyll-a content, the primary photosynthetic pigment, along with a corresponding decline in photosynthetic efficiency.^39,40^ The impact of nAg on the algal photosystem of *C. reinhardtii* and *Euglena gracilis* is suggested to arise from dissolved silver coexisting with the nAg.^32,41^ Ag^+^ were shown to replace copper ions (Cu^+^) in the catalytic centers of electron transport chain components, or to complex with thiol (–SH) groups of enzymes involved in chlorophyll biosynthesis.^42^ In our study, exposure to 0.3 mg L^−1^ nAg resulted in a significant impairment of photosynthetic yield, whereas exposure to 0.01 mg L^−1^ Ag^+^, which corresponds to the equivalent dissolved silver concentration after 2 h, did not induce any detectable reduction in photosynthetic performance (Fig. 3A). Given that Ag^+^ is recognized as the primary agent of Ag-induced phototoxicity, this discrepancy suggests that the intracellular dissolution of nAg may play a critical role. Indeed, intracellular biotransformation of nAg *via* dissolution has been previously reported in the green alga *C. reinhardtii*.^43^

NPQ in *C. meneghiniana* cells significantly increased for all exposure conditions except for the highest tested Ag^+^ exposure (0.02 mg L^−1^) (Fig. 3B). Diatoms possess an efficient mechanism to dissipate excess excitation energy as heat to protect PSII from damage, which is illustrated by NPQ of chlorophyll a fluorescence.^44,45^ The increase in NPQ signifies an activation of photoprotective mechanisms by the organism under exposure to Ag^+^ and nAg.^46,47^ In the literature, reported NPQ responses to nAg vary widely and appear to depend on size, surface coating, test organism, and exposure duration. For example, a twofold increase in NPQ was observed in the alga *P. malhamensis* following a 2 h exposure to 1 mg L^−1^ nAg (20 nm).^17^ In contrast, in the diatom *Skeletonema costatum*, exposure to increasing concentrations of nAg (0.5–10 mg L^−1^, 10 nm) over 24 h resulted in a decrease in NPQ.^40^ In cases of prolonged or severe stress, such as those described in *S. costatum*, cells may lose the capacity to activate protective mechanisms like NPQ, unlike the short-term exposures used in our study. Similarly, in our study, the lack of activation of the NPQ photoprotective mechanism under the highest concentration of Ag^+^, 0.02 mg L^−1^, may be attributed to the higher toxicity of Ag^+^ toward the photosynthetic apparatus compared to nAg.

Significant accumulation of ROS was observed here only in cells exposed to the lowest nAg concentration, 0.1 mg L^−1^ (Fig. 3C). ROS generation is a well-documented toxicity mechanism associated with nAg.^37,48^ Enhanced ROS production has been reported in various phytoplankton species, including the green freshwater alga *C. vulgaris*, marine alga *Dunaliella tertiolecta* (50 nm, 1–10 mg L^−1^, 24 h),^49^*C. reinhardtii* (45 nm, 1 mg L^−1^, 48 h),^50^ and the marine diatom *S. costatum* (10 nm, 0.5–50 mg L^−1^, 24 h).^40^ The accumulation of ROS can initiate cellular defense mechanisms, such as the activation of NPQ, which was observed under the 0.1 mg L^−1^ nAg treatment in our study (Fig. 3B). However, exposure to the highest nAg concentration (0.3 mg L^−1^) did not result in any significant intracellular ROS accumulation in *C. meneghiniana* (Fig. 3C). Similarly, no increase in intracellular ROS levels was found for *C. meneghiniana* exposed to a higher nAg concentration for a longer duration (20 nm, 0.5 mg L^−1^, 24 h).^51^ This lack of ROS may be attributed to extensive cellular damage at higher nAg concentrations, leading to reduced metabolic activity and consequently diminished ROS production. Indeed, in our study, *C. meneghiniana* exposed to 0.3 mg L^−1^ nAg exhibited approximately 4% higher membrane damage and significantly reduced photosynthetic yield compared to control cells after 2 h exposure (Fig. S2 and 3A). Similarly, no ROS accumulation was observed in cells exposed to Ag^+^, which could be linked to its higher direct toxicity or differences in the mechanisms of ROS induction between the two silver forms (Fig. 3C). For instance, *S. costatum* accumulated ROS under nAg exposure, while exposure to Ag^+^ led to a slight reduction in ROS levels, further highlighting the distinct modes of action of nAg and Ag^+^.^40^ Notably, the absence of ROS accumulation for the highest Ag^+^ exposure (0.02 mg L^−1^) was accompanied with significantly reduced photosynthetic activity compared to control cells highlighting acute toxicity (Fig. 3A). However, membrane permeability in Ag^+^-treated *C. meneghiniana* cells remained comparable to that of control cells (Fig. S2), suggesting limited membrane disruption under the conditions tested.

Generation of ROS is known to compromise the structural integrity of organelles involved in energy production, such as chloroplasts and mitochondria, thereby impairing ATP generation.^52,53^ Reduced ATP availability is often linked to diminished carbon fixation capacity in photosynthetic organisms.^54,55^ Carbonic anhydrase (CA), a key enzyme in this process, catalyzes the reversible hydration of CO_2_ and facilitates the uptake of inorganic carbon by cells.^56^ In our study, total CA activity in *C. meneghiniana* remained largely unaffected across most Ag^+^ and nAg treatments. However, a significant increase in CA activity was observed under the lowest nAg exposure condition (0.1 mg L^−1^) (Fig. 3D). Although CA activity is generally reported to decrease or become inhibited under metal stress conditions involving Ag^+^, Hg^2+^, or Pb^2+^,^57^ moderate stress has also been shown to upregulate CA activity in some photosynthetic organisms.^52,58^ For example, in the green alga *C. reinhardtii*, short-term exposure to a low concentration (0.001 mg L^−1^) of Ag^+^ led to an upregulation of CA expression at the transcriptomic level.^52^ In this study, the simultaneous increase in ROS levels, NPQ, and CA activity under short-term exposure to 0.1 mg L^−1^ nAg may reflect the activation of cellular defense mechanisms in *C. meneghiniana* in response to mild nanoparticle-induced stress.

### 3.4. Metabolic perturbations upon nAg and Ag^+^ exposure

#### 3.4.1. General patterns of metabolic responses to nAg and Ag^+^

**Fig. 4:**
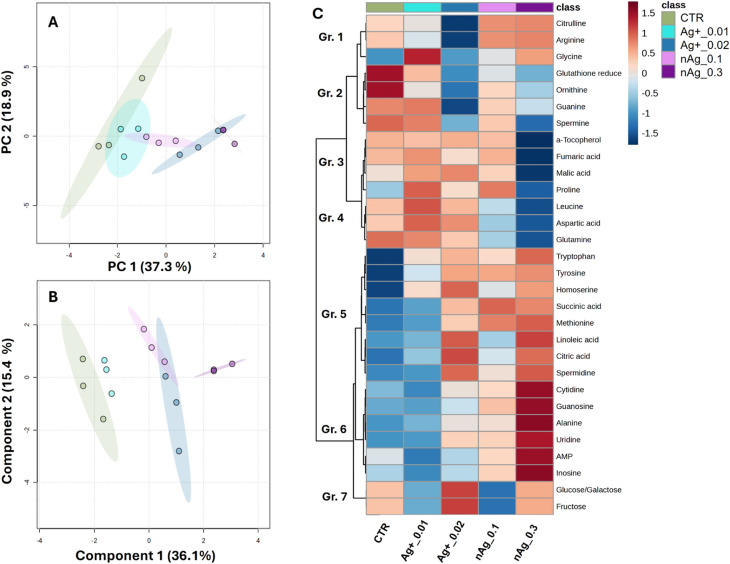
(A) Principal component analysis (PCA) and (B) partial least-squares discriminant analysis (PLS-DA) score plots of metabolic profiles of *C. meneghiniana* exposed to dissolved silver (Ag^+^; 0.01 and 0.02 mg L^−1^) and nanoparticulate silver (nAg; 0.1 and 0.3 mg L^−1^). (C) Clustering of responsive metabolites displayed as a heatmap.

In the metabolomics analysis, a total of 101 metabolites were considered including major metabolic groups such as amino acids, antioxidants, organic acids/phenolics, nucleobases/sides/tides, sugars/sugar alcohols, polyamines, pigments and fatty acids. Sixty metabolites were detected and quantified across all Ag treatments and control samples. Unsupervised principal component analysis (PCA) demonstrated separation of all treatments from the control group along principal component 1, describing 37.3% of the total variance, except for the lowest Ag^+^ treatment (0.01 mg L^−1^) (Fig. 4A). Furthermore, supervised partial least squares discriminant analysis (PLS-DA) established a good separation between all treatments, except for 0.02 mg L^−1^ Ag^+^ and 0.1 mg L^−1^ nAg, which did not exhibit clear separation (Fig. 4B). Addition of a third component improved discrimination between these two treatment groups, yielding a statistically valid PLS-DA model (Fig. S3). These two treatment groups showed comparable intracellular Ag levels, whereas their adsorbed Ag differed significantly (Fig. 2). Subsequently, based on ANOVA (*p* < 0.05, Table S3) and PLS-DA VIP score analysis (VIP > 1; Fig. S4) based on the validated three-component model, a total of 30 responsive metabolites were detected among all treatments.

The cluster analysis of the responsive metabolites revealed seven major metabolic groups (Fig. 4C). The first group exhibited a dual effect, where three metabolites (3AA) were depleted in Ag^+^ treatments but accumulated in nAg treatments in a concentration-dependent manner. The second group consisted of four metabolites (1AO, 1AA, 1 nucleotide and 1 PA) that were depleted in a concentration-dependent manner for both Ag^+^ and nAg treatments. The third group comprised four metabolites (1AO, 2 OA and 1AA), which were significantly depleted only in the highest nAg treatment (0.3 mg L^−1^). Similarly, the fourth group displayed a dual effect, with three metabolites (3AA) accumulating in Ag^+^ treatments but depleting in nAg treatments. The fifth group included eight metabolites (4AA, 2 OA, 1FA and 1PA) that accumulated in a concentration-dependent manner across all conditions. In the sixth group, six metabolites (5 nucleotide/side, 1AA) were identified as accumulating in a concentration-dependent manner for nAg treatments, whereas the trend appeared to be less clear for Ag^+^. Finally, the seventh group consisted of 2 sugars, with no clear accumulation/depletion pattern. Overall, the metabolic alterations were more pronounced in nAg treatments and followed a concentration-dependent trend for both Ag^+^ and nAg. Pathway analysis revealed eighteen significantly altered biochemical pathways (threshold < 0.1) (Fig. S5), highlighting that even 2 h exposure to low concentrations of nAg and Ag^+^ led to broad metabolic reprogramming in *C. meneghiniana*, underscoring their potential as sensitive biomarkers for early nanoparticle toxicity detection.

#### 3.4.2. Key metabolic alterations in *C. meneghiniana* exposed to nAg and Ag^+^

##### Amino acid and polyamine metabolism

**Fig. 5:**
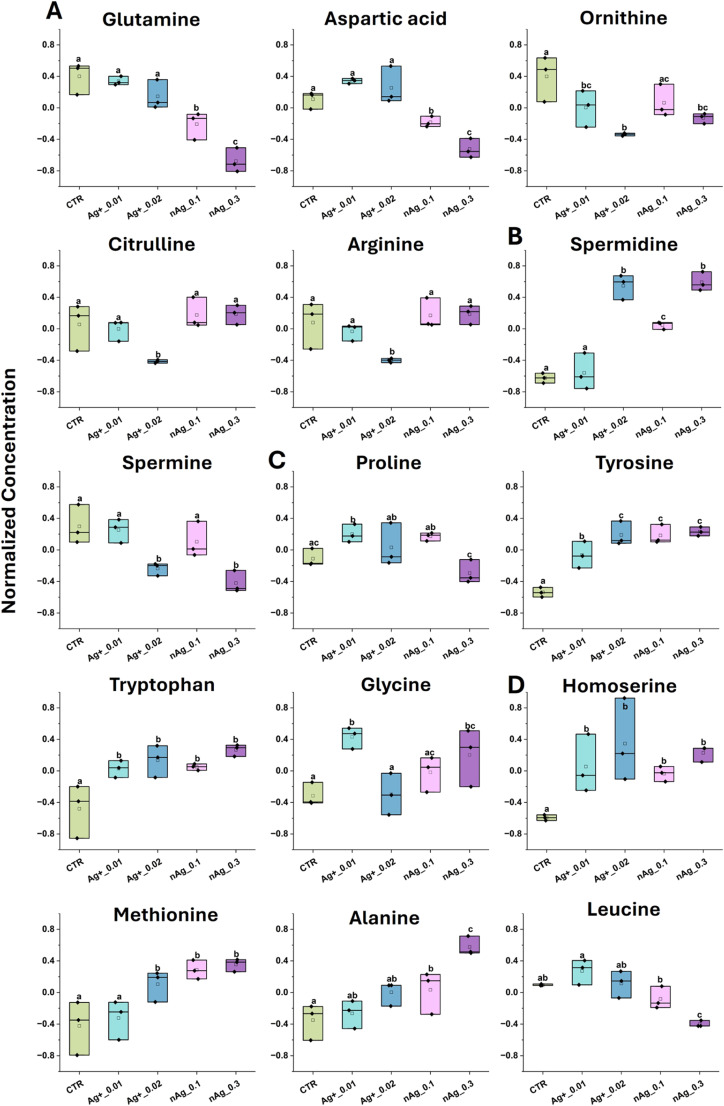
Boxplots showing changes in responsive amino acids and polyamines in *C. meneghiniana* after 2-h exposure to two concentrations of dissolved silver (Ag^+^; 0.01 and 0.02 mg L^−1^) and nanoparticulate silver (nAg; 0.1 and 0.3 mg L^−1^). Only metabolites meeting the criteria with ANOVA *p* < 0.05 and VIP > 1 are presented. Metabolites are grouped as follows: (A) nitrogen metabolism and urea cycle, (B) polyamine metabolism and implications for cellular structure, (C) stress-response and cellular protection and (D) metabolic reallocation and protein synthesis. Different letters indicate statistically significant differences between conditions (one-way ANOVA followed by Fisher's LSD *post hoc* test). Boxes represent the interquartile range (25th–75th percentiles), with the median shown as a solid line. Means are indicated by open squares, and individual data points are displayed as filled diamonds.

Amino acid metabolism was markedly impacted in *C. meneghiniana* following 2 h exposure to both nAg and Ag^+^, with 15 AAs and PAs showing significant alterations (*p* < 0.05). AAs are essential metabolites, serving not only as the molecular building blocks of proteins but also as precursors for the biosynthesis of other metabolites involved in critical biological processes, such as growth.^59,60^ In diatoms, AA biosynthesis is intricately linked to central carbon, nitrogen, and sulfur metabolism.^61^ Among the 15 responsive AAs, 8 showed accumulation, while 7 were depleted following exposure to nAg, Ag^+^, or both (Fig. 5). The AAs and PAs that were significantly accumulated (*p* < 0.05) including homoserine, methionine, spermidine, tryptophan, tyrosine, alanine, glycine, and proline. Meanwhile levels of ornithine, citrulline, arginine, spermine, glutamine, aspartic acid and leucine were significantly depleted (*p* < 0.05).

###### Disruption of the nitrogen metabolism and urea cycle

Silver exposure, particularly nAg, strongly affected amino acids involved in nitrogen assimilation and urea cycle metabolism. Glutamine, a central amino acid in nitrogen assimilation, was depleted under nAg exposure, but not under Ag^+^ exposure (Fig. 5A). In diatoms, glutamine is formed by incorporation of ammonia *via* glutamine synthetase^61^ and also serves as a precursor for nucleotide biosynthesis. Its depletion therefore suggests impaired nitrogen assimilation and reduced nucleotide synthesis, indicating intracellular nitrogen imbalance under nAg stress. Similar glutamine depletion has been reported in *P. malhamensis* under Ag exposure,^17^ although responses to Ag^+^ appear species-specific.

Aspartic acid showed a similar pattern, decreasing under nAg but not Ag^+^ exposure (Fig. 5A).

Derived from oxaloacetate, a key intermediate of the TCA cycle, aspartate links nitrogen assimilation to central carbon metabolism and serves as a precursor for several AAs' biosynthesis, including threonine, methionine, and lysine.^62^ Hence, its depletion indicates that nAg more strongly disrupts both nitrogen assimilation and central carbon metabolism in *C. meneghiniana* compared to Ag^+^. This is consistent with the reported decrease in the relative abundance of aspartic acid in *C. vulgaris* under nAg exposure.^11^

Ornithine, a key intermediate in arginine biosynthesis linked to the urea cycle,^61^ decreased in a concentration-dependent manner in *C. meneghiniana* under exposure to both nAg and Ag^+^ (Fig. 5A). As a precursor for multiple downstream pathways, its depletion suggests altered metabolic flux within nitrogen metabolism. Consistently, citrulline and arginine, derived from ornithine, were specifically depleted at the highest Ag^+^ concentration (0.02 mg L^−1^) (Fig. 5A), suggesting disruption of the urea cycle. In diatoms, this pathway supports intracellular reallocation of nitrogen and inorganic carbon, processes essential for growth and adaptation to nutrient limitations.^61,63^

###### Polyamine metabolism and implications for cellular structure

Perturbation of nitrogen metabolism extended to polyamine biosynthesis, with potential consequences for cellular structure. The polyamine spermidine accumulated in a concentration-dependent manner in *C. meneghiniana* under both nAg and Ag^+^ exposure (Fig. 5B). As a key growth regulator, spermidine supports photosynthetic pigment synthesis, cell proliferation, membrane integrity^64^ and ROS scavenging under stress.^65^ Its accumulation may also affect frustule formation, given its role in diatom silicification.^66^ Consistently, spermine was depleted in *C. meneghiniana* under 0.02 mg L^−1^ Ag^+^ and 0.3 mg L^−1^ nAg (Fig. 5B). As a downstream product of spermidine,^64^ its decrease, alongside spermidine accumulation, suggests impaired conversion and disruption of PA homeostasis. This is supported by a marked increase in the spermidine/spermine ratio: 8.33 ± 1.85 in control cells, compared to 22.70 ± 4.88, 67.78 ± 16.32, and 55.29 ± 11.29 in Ag^+^ 0.01, 0.02 mg L^−1^ and nAg 0.3 mg L^−1^ treatments, respectively. Together, these results point to Ag-induced perturbation of the urea cycle, affecting downstream polyamine synthesis and potentially altering frustule formation.

###### Activation of stress-response and protective amino acids

In parallel, several amino acids associated with stress response and redox homeostasis accumulated under silver exposure. Proline, a well-known antioxidant involved in osmoregulation and redox homeostasis,^67^ also contributes to protein stability and membrane integrity.^68^ In this study, proline increased only at the lowest Ag^+^ concentration (Ag^+^_0.01) (Fig. 5C). In contrast, proline accumulation under nAg exposure has been reported in *C. pyrenoidosa* and *P. malhamensis*,^13,17^ highlighting species-specific metabolic responses to Ag stress.

Tyrosine and tryptophan, two aromatic amino acids synthesized *via* the shikimate pathway, accumulated under both nAg and Ag^+^ exposures (Fig. 5C). These amino acids support protein integrity and key cellular processes, including growth, division, DNA replication, and defense mechanisms.^69^ They further act as precursors for essential metabolites such as tocopherols and components of photosynthetic electron transport.^70,71^ Consistent with these functions, combined physiological and metabolomic analyses revealed compromised photosystem performance in *C. meneghiniana*, with α-tocopherol significantly affected under silver exposure (Fig. 3A and B and 4). Their accumulation may therefore reflect shifts in intracellular energy metabolism and activation of defense mechanisms against Ag-induced stress. While tyrosine accumulation aligns with elevated levels reported in *C. pyrenoidosa* and *N. sphaeroides* under nAg and Ag^+^ exposure,^13,16^ contrasting trends for tryptophan in *C. vulgaris* and *P. malhamensis*^12,17^ further highlight species-specific metabolic responses to Ag forms.

Glycine also accumulated under both nAg and Ag^+^ treatments, although without a clear concentration-dependent pattern (Fig. 5C). As a product of photorespiration, glycine may reflect the activation of this pathway as an alternative energy sink under oxidative stress and reduced CO_2_ assimilation, helping to mitigate photoinhibition.^72^ This is consistent with physiological findings in *C. meneghiniana*, where nAg exposure disrupted photosynthetic performance and impaired CA activity (Fig. 3). Together, these changes indicate a shift toward protective metabolic pathways under silver exposure.

###### Metabolic reallocation and implications for protein synthesis

Several amino acids further indicated metabolic reallocation and altered protein dynamics under Ag stress. Homoserine and methionine increased in a concentration-dependent manner under both nAg and Ag^+^ exposure (Fig. 5D). In diatoms, synthetized from aspartate, homoserine serves as a precursor to methionine.^61^ Methionine contributes to metal detoxification, largely due to its sulfhydryl group (–SH), which exhibits high affinity for Ag^+^.^73,74^ Beyond its detoxification function, methionine is a major component of transmembrane proteins involved in Cu^+^ transport, which can also be exploited by Ag^+^.^75,76^ In a previous study involving *C. meneghiniana*, methionine accumulation was observed under mercury exposure, known for its strong affinity to methionine.^77^ Collectively, these findings suggest that the accumulation of homoserine and methionine may reflect enhanced Ag^+^ transport, activation of defense pathways or reduced protein synthesis.

Leucine, a branched-chain amino acid, decreased only under 0.3 mg L^−1^ nAg exposure (Fig. 5D). Diatoms secrete extracellular polymeric substances (EPS), enriched in proteins, in response to stress.^23,78^ The extracellular matrix of diatom *Phaeodactylum tricornutum*, contains leucine-rich repeat (LRR) proteins, involved in adhesion.^79^ Combined with evidence of enhanced EPS secretion in *C. meneghiniana* under nAg exposure,^51^ this depletion may reflect leucine allocation to EPS-associated protein synthesis during nAg stress adaptation.

Alanine, derived from pyruvate,^80^ accumulated exclusively under nAg exposure, indicating a shift in cellular metabolism (Fig. 5D). Its accumulation under metal stress has been linked to hypoxia-like conditions and metabolic reorganization, as reported in *C. vulgaris* under Cd and Pb stress.^81,82^ Together, these changes indicate a redistribution of metabolic resources under silver stress, affecting protein synthesis and energy metabolism.

Overall, amino acid profiling indicates extensive metabolic reprogramming in *C. meneghiniana* under silver exposure, more pronounced for nAg than Ag^+^, with key nitrogen metabolism-related amino acids, glutamine and aspartic acid, altered exclusively under nAg exposure. Disruption of nitrogen metabolism, including urea cycle intermediates, suggests impaired nitrogen assimilation and reallocation. Concurrent accumulation of stress-related amino acids and polyamines, alongside altered polyamine balance, reflects activation of defense pathways and urea cycle perturbation. Together with evidence for resource reallocation and impaired photosynthesis, these changes highlight an integrated response linking nitrogen metabolism, stress adaptation, and cellular function under Ag exposure.

##### Carboxylic acid metabolism

**Fig. 6:**
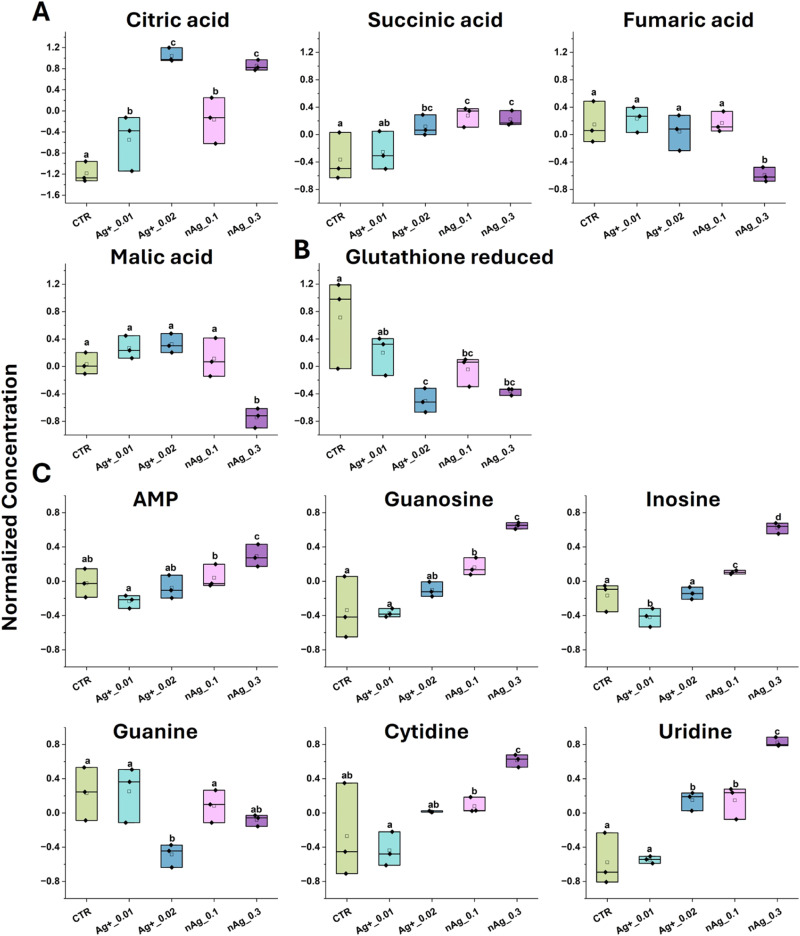
Boxplots showing changes in responsive amino acids and polyamines in *C. meneghiniana* after 2-hour exposure to two concentrations of dissolved silver (Ag^+^; 0.01 and 0.02 mg L^−1^) and nanoparticulate silver (nAg; 0.1 and 0.3 mg L^−1^). Only metabolites meeting the criteria with ANOVA *p* < 0.05 and VIP > 1 are presented. (A) Significantly altered organic acids, (B) reduced glutathione and (C) nucleobase/side/tide. Different letters indicate statistically significant differences between conditions (one-way ANOVA followed by Fisher's LSD *post hoc* test). Boxes represent the interquartile range (25th–75th percentiles), with the median shown as a solid line. Means are indicated by open squares, and individual data points are displayed as filled diamonds.

The TCA cycle, at the core of cellular respiration, was impacted, as reflected by significant changes in the relative abundance of four key organic acids: citric, succinic, fumaric, and malic acid (Fig. 6A). Citric acid exhibited a concentration-dependent accumulation across all Ag treatments, while succinic acid increased only under 0.01 mg L^−1^ Ag^+^ treatment (Fig. 6A). In contrast, fumaric and malic acid were depleted only under 0.3 mg L^−1^ nAg exposure, highlighting the specific sensitivity of the TCA cycle to nAg (Fig. 6A). In diatoms, the TCA cycle is known to be tightly regulated by the ornithine–urea and glutamine synthetase/glutamate synthase cycles, both of which were disrupted in this study.^63,83^ Malic acid plays a role in diatom-specific photorespiratory pathways, further supporting its sensitivity to nAg stress.^84^

These findings are consistent with previous studies reporting TCA cycle alterations under metal exposure, although responses vary across species and conditions.^11,17,77^ Altogether, our results highlight the specific impact of nAg on key respiratory metabolisms of *C. meneghiniana*, particularly through disruption of the TCA cycle.

##### Glutathione metabolism

Reduced glutathione (GSH), a key intracellular antioxidant, was depleted under short-term exposure to nAg and Ag^+^ in *C. meneghiniana* (Fig. 6B), contrasting with studies reporting increased GSH under metal stress.^26,77,85^ This depletion may reflect its consumption during phytochelatin (PC) synthesis, as observed in other alga under metal exposure, where increased PC production coincides with oxidative stress.^86,87^ PCs, in turn, play a central role in metal detoxification by chelating excess intracellular metals. Alternatively, GSH depletion may result from its utilization in antioxidant defense, consistent with elevated ROS levels under nAg exposure (Fig. 3C), although no increase in glutathione peroxidase (GPx) activity was detected (Fig. S6).^88,89^ Another possibility is its involvement in alternative detoxification pathways, such as conjugation with spermidine to form glutathionylspermidine (GSP), as reported in other organisms.^90^ Given the observed perturbation of polyamine homeostasis (Fig. 5), a similar mechanism may occur in diatoms, although this remains to be confirmed.

Together, these findings suggest that GSH depletion reflects an increased demand for detoxification and antioxidant defenses under silver induced stress.

##### Nucleobase, nucleotide and nucleoside metabolism

Among the 15 nucleobases/tides/sides analyzed, 14 were detected across all conditions with six showing significant alterations in response to Ag^+^, nAg, or both forms (*p* < 0.05) (Fig. 6C). Purine metabolism was more strongly affected by nAg, with AMP and guanosine accumulating exclusively under nAg exposure, while inosine showed opposite trends (increase under nAg, decrease under Ag^+^) and the relative abundance of guanine remained largely unchanged except at 0.02 mg L^−1^ Ag^+^. In contrast, pyrimidine nucleosides (cytidine and uridine) accumulated under both nAg forms, with a more pronounced increase under nAg.

The accumulation of nucleobases and nucleosides may reflect enhanced DNA damage and repair processes under silver stress,^91^ consistent with the known genotoxic effects of nAg *via* direct DNA interaction or ROS generation.^92^ In contrast, AMP accumulation may indicate *de novo* nucleotide synthesis, supported by depletion of glutamine and aspartate under nAg exposure (Fig. 5B), suggesting their utilization in purine biosynthesis.

Overall, these results indicate that nucleotide metabolism is reprogrammed under Ag stress, particularly nAg, reflecting both genotoxic damage and increased biosynthetic demand linked to nitrogen reallocation.

#### 3.4.3. nAg exposure drives specific metabolic responses in *C. meneghiniana*

To gain further understanding of the specific metabolic responses induced by nAg exposure in *C. meneghiniana*, a data analysis was performed comparing the metabolic profiles of cells exposed to 0.3 mg L^−1^ nAg and 0.01 mg L^−1^ Ag^+^, representing dissolved silver concentration in nAg suspensions. PCA demonstrated separation of nAg_0.3 from the control group along principal component 1, describing 46.8% of the total variance, while the group corresponding to Ag^+^ treatment was not separated from the control group (Fig. S7). These results indicate that nAg exposure induces a distinct metabolic response in *C. meneghiniana*, while Ag^+^ at an equivalent dissolved concentration elicits only limited metabolic alterations, largely overlapping with the non-exposed control.

**Fig. 7:**
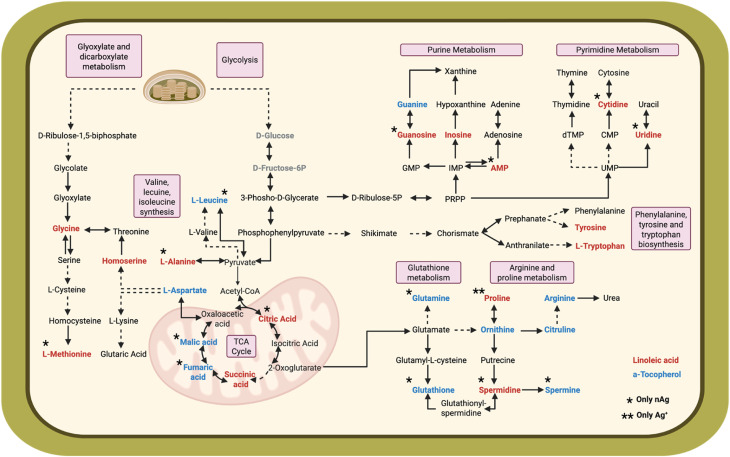
Conceptual model of metabolic changes in *C. meneghiniana* after 2 h exposure to dissolved silver (Ag^+^; 0.01 and 0.02 mg L^−1^) and nanoparticulate silver (nAg; 0.1 and 0.3 mg L^−1^). Only metabolites meeting the criteria with ANOVA *p* < 0.05 and VIP > 1 are presented. Metabolite depletions are indicated in blue, accumulations in red, and unclear trends in grey. Ag species–specific changes were identified by comparative analysis between nAg_0.3 and Ag^+^_0.01, with changes unique to nAg denoted by * and those unique to Ag^+^ denoted by **.

This analysis yielded 17 responsive metabolites (*p* < 0.05) (Table S4). Among the responsive metabolites identified, 5 were shared between treatments, while 11 were significantly altered exclusively under exposure to nAg (Fig. 7 and S7). In contrast, only 1 metabolite was significantly affected by Ag^+^ treatment (Fig. 7 and S7). Among nAg responsive metabolites, several TCA cycle intermediates (citric, malic, and fumaric acid) and related amino acids (alanine, leucine) were affected, suggesting a stronger impact of nAg on the central carbon metabolism (Fig. 7). PA-related metabolites, including glutamine, spermidine, and spermine, were also altered only under nAg, consistent with perturbation of nitrogen-linked metabolic pathways (Fig. 7). In addition, changes in purine (AMP, guanosine) and pyrimidine (uridine) metabolites under nAg exposure further indicate alterations in the nucleotide metabolism (Fig. 7).

In contrast, proline was the only metabolite significantly affected exclusively by Ag^+^ (Fig. 7 and S7), consistent with its role in stress response and suggesting a more limited or targeted metabolic adjustment under ionic silver exposure. Metabolites commonly affected by both silver forms were predominantly amino acids, including aspartic acid, homoserine, tyrosine, and tryptophan, highlighting the shared impact of nAg and Ag^+^ on the amino acid metabolism (Fig. 7 and S7).

The higher number of affected metabolites observed under nAg exposure may be related to nanoparticle-specific effects, such as intracellular dissolution and sustained Ag^+^ release.^43^ These alterations likely reflect a combination of metabolic disruption, compensatory responses, and stress induced metabolic reallocation rather than a single underlaying mechanism. Overall, these results suggest that nAg induces more extensive metabolic changes than Ag^+^ at equivalent dissolved concentrations.

## 4. Conclusions

Our study provides the first evidence of metabolic perturbations in the diatom *C. meneghiniana* following short-term exposure to both nAg and Ag^+^. By integrating metabolomics with conventional physiological analyses, we identified concentration- and silver form-specific toxicity responses. Both silver forms disrupted amino acid, polyamine, TCA cycle, glutathione, purine, and pyrimidine metabolism, though the extent and nature of these effects differed between forms. Notably, the metabolic alterations appeared to be concentration-dependent for each Ag species, as increasing exposure concentrations led to higher intracellular Ag levels. The nanoparticulate form particularly exerted distinct effects on the TCA cycle, glutathione metabolism, and polyamine production, highlighting a unique nanoparticle-driven contribution to metabolic disruption in diatom *C. meneghiniana*. Consistent with these metabolomic shifts, nAg also had a more pronounced impact on diatom physiology, as evidenced by enhanced ROS generation and altered CA activity. Our study demonstrates that metabolomics is a powerful approach to uncover early metabolic responses of diatoms to different forms of silver, while specifically revealing the unique role of the nanoparticulate form in driving metabolic reprogramming.

## Author contributions

Arin Kantarciyan: writing – original draft, visualization, methodology, investigation, formal analysis, conceptualization. Inés Segovia-Campos: writing – review & editing, methodology, investigation, conceptualization. Matea Marelja: writing – review & editing, methodology, investigation. Rocco Gasco: writing – review & editing, methodology, investigation. Weiwei Li: methodology, investigation. Arturo A. Keller: writing – review & editing, validation, supervision, conceptualization. Vera I. Slaveykova: writing – review & editing, validation, supervision, project administration, funding acquisition, conceptualization.

## Conflicts of interest

The authors declare that they have no known competing financial interests or personal relationships that could have appeared to influence the work reported in this paper.

## Supplementary Material

EN-013-D6EN00326E-s001

## Data Availability

Data of this article including dataset for nAg characterization, bioassays and metabolomic analysis are available on research archive YARETA at https://doi.org/10.26037/yareta:dplu2rc3trf7fh2bwtejdsw6lm. Supplementary information (SI) is available. See DOI: https://doi.org/10.1039/d6en00326e.
