# Supplementary material for: Metabolomic profiling of diatoms reveals distinct impacts of silver nanoparticles and ions
Source: Environ Sci Nano. 2026 Jun 4;13(7):3028–43. doi: 10.1039/d6en00326e (PMC13273387; doi:10.1039/d6en00326e)
Supplement: EN-013-D6EN00326E-s001 [file EN-013-D6EN00326E-s001.pdf]

Supplementary Information (SI) for Environmental Science: Nano.
This journal is © The Royal Society of Chemistry 2026

Supplementary Information

Metabolomic Profiling of Diatoms Reveals Distinct Impacts of Silver Nanoparticles and Ions

Arin Kantarciyan [1], Inés Segovia-Campos [1], Matea Marelja[1], Rocco Gasco[1], Weiwei Li[2], Arturo A. Keller[2], and Vera I. Slaveykova[1, *]

[1] University of Geneva, Faculty of Sciences, Department F.-A. Forel for Environmental and Aquatic Sciences, Environmental biogeochemistry and ecotoxicology, Bvd Carl-Vogt 66, 1205-Geneva Switzerland

[2] Bren School of Environmental Science & Management, University of California, Santa Barbara, CA 93106-5131, United States

* *Corresponding author*

Phone: +41 22 379 0335

E-mail: vera.slaveykova@unige.ch

Table of Contents

1. Quantification of intracellular and adsorbed silver fractions..................................3

2. Photosynthetic activity analysis...3

3. Membrane permeability..4

4. Total cellular reactive oxygen species (ROS)..4

5. Total Carbonic anhydrase (CA) activity analysis 5

6. Analysis of metabolomics data with Metaboanalyst 6.0 6

Table S1. Synthetic freshwater medium (SFM)+ Si Composition...8

Table S2. List of metabolites and the MS parameters for LC-MS targeted metabolomics....9

Table S3. Key features identified by One-way ANOVA and Fisher's post-hoc analysis in *C. meneghiniana* 14

Table S4. Key features identified by One-way ANOVA and Fisher's post-hoc analysis in *C. meneghiniana* 16

Table S5. Detailed results from the pathway analysis... 17

Figure S1. *C. meneghiniana* growth inhibition curve 19

Figure S2. Percentage of PI-stained *C. meneghiniana* cells 20

Figure S3. Three-component validated partial least squares discriminant analysis (PLS-DA) model assessed by cross validation and permutation testing. (A) Score plot showing sample distribution based on first three components. (B) Classification performance of PLS-DA models using varying numbers of components. The asterisk indicates the best classifier. (C) PLS-DA model validation based on separation distance. The p value based on permutation is p=0.002 (2/1000)... 21

Figure S4. Variable Importance in Projection (VIP) scores from three-component PLS-DA model, discriminating between the control (CTR), two concentrations of dissolved silver (Ag+_0.01: 0.01 mg L^{-1} Ag and Ag+_0.02: 0.02 mg L^{-1} Ag), and two concentrations of nanoparticulate silver (nAg_0.1: 0.1 mg L^{-1} Ag and nAg_0.3: 0.3 mg L^{-1} Ag). 22

Figure S5. Results of pathway analysis 23

Figure S6. Glutathione peroxidase activity in *C. meneghiniana* 25

Figure S7. Comparative metabolomic responses of *C. meneghiniana* exposed to nanoparticulate and equivalent dissolved silver concentration. 26

1. Quantification of intracellular and adsorbed silver fractions

Twenty mL of cell aliquots were harvested and rinsed once with fresh SFM exposure medium. Then, collected pellets were rinsed with 0.5 mmol L^{-1} hydrogen peroxide (H_2O_2) (Merck KGaA, Germany) followed by an extracting step where cells were incubated with 1 mmol L^{-1} D-penicillamine (D-pen) (Alfa Aesar). H_2O_2 and D-penicillamine solutions (no cells) were collected and acidified to 2% HNO_3 (v:v) to quantify the adsorbed Ag concentrations, following previously optimized procedure in our laboratory [1]. The sum of these two fractions was considered as the total adsorbed content. Then, the pellets were rinsed one last time with clean SFM exposure medium and digested with 160 µL 68% HNO_3 and 40 µL 30% H_2O_2 at 90°C for 2 h. Subsequently, the fractions were analyzed for total silver concentration by ICP-MS.

2. Photosynthetic activity analysis

Information on the state of photosystem II (PSII) of *C. meneghiniana* cells was obtained using FluorCam 800MF in pulse amplitude modulated mode. Cell aliquots of 1 mL were harvested and transferred to a 24-well plate, then dark-acclimated for 30 minutes. Following acclimation, cells were analyzed under a secondary actinic light source (Actinic light 2). The minimum fluorescence in the dark-adapted state (F_0) was measured over 30 seconds. Pulse duration was set to 800 ms, with a dark pause of 20 seconds between pulses. The total exposure time to actinic light 2 was 900 seconds, followed by a relaxation interval of 180 seconds. A total of 10 pulses were applied during the measurement. The intensity of actinic light was maintained at 90 µmol s^{-1} m^{-2}, corresponding to the light conditions in the cell growth chamber. Fluorescence data were analyzed using FluorCam7 software (Drásov, Czech Republic).

3. Membrane permeability

Membrane permeability was assessed using flow cytometry as described elsewhere [1]. Briefly, propidium iodide (PI) (Sigma-Aldrich, Buchs, Switzerland) was dissolved in DMSO and added to cell suspensions ($1x10^9$ cells L^{-1}) in SFM to a final concentration of 0.7 µmol L^{-1}. Then the mixture was incubated for 30 min in dark and room temperature and analyzed via flow cytometry (BD Accuri C6, BD Biosciences, San Jose, CA, USA). Unexposed cells were used as negative controls whereas cells heated to 90 °C for 15min were used as positive controls. Gating strategies for flow cytometry analysis could be found elsewhere [1].

4. Total cellular reactive oxygen species (ROS)

Total intracellular reactive oxygen species (ROS) levels were quantified using the fluorescent probe 5-(and-6)-chloromethyl-2',7'-dichlorodihydrofluorescein diacetate (CM-H_2DCFDA) (MedChemExpress LLC, Monmouth Junction, USA) following the method of [2] with adaptation to diatom *C. meneghiniana*. A stock solution of 9.62 µM CM-H_2DCFDA was prepared by dissolving the probe in DMSO. Briefly, 20 mL of cell culture (approximately 6 × 10^8 cells L^{-1}) was harvested by centrifugation at 4121 × *g* for 10 min at 4°C, and the pellet was resuspended in 0.5 mL of cold phosphate-buffered saline (PBS; pH 7.4). Cells were homogenized on ice using a Sonics Vibra-Cell sonicator (Sonics & Materials Inc., Connecticut, USA) at 60% amplitude for 2 minutes with alternating 5-second pulses and 2-second pauses.

The homogenates were then centrifuged at 10,000 × *g* for 10 minutes at 4°C to remove cell debris and wall fragments. For ROS quantification, 10 µL of each supernatant was transferred into a 96-well microplate, followed by the addition of 70 µL PBS and 10 µL CM-H_2DCFDA. Each condition was tested with four biological replicates, each analyzed in technical triplicate. Negative controls consisted of samples without dye addition, while positive controls included 2 µL of 0.019 mol L^{-1} H_2O_2 (prepared in Milli-Q water), 88 µL PBS, and 10 µL CM-H_2DCFDA.

Fluorescence was recorded every 2 minutes for 90 minutes using a BioTek Synergy H1 microplate reader (BioTek Instruments Inc., Winooski, USA) at an excitation wavelength of 495 nm and emission of 530 nm. For data analysis, the first 4 minutes of readings were excluded to account for dye stabilization.

5. Total Carbonic anhydrase (CA) activity analysis

Total carbonic anhydrase (CA) activity was measured using an electrometric technique based on pH variations, following the Wilbur-Anderson assay with minor modifications [3, 4]. Briefly, a cell aliquot of 19 mL was sampled, rinsed once with fresh SFM exposure medium, and resuspended in 900 µL extraction buffer consisting of 20 mmol L^{-1} TRIS-HCl (pH 8.3) and 2 mmol L^{-1} dithiothreitol (DTT) (ROTH AG, Arlesheim, Switzerland). Cells extracts were obtained via homogenization on ice using a Sonics Vibra-Cell sonicator (Sonics & Materials Inc., Connecticut, USA) at 60% amplitude for 2 minutes with alternating 5-second pulses and 2-second pauses.

For the measurements, 600 µL of cell extract was diluted in 8 mL of extraction buffer and kept on ice. Then, 4 mL of ice-cold MQ water saturated with CO_2 was added, and the time required for the pH to drop from 8.3 to 7.9 was recorded. MQ water was pre-saturated with CO_2 by bubbling on ice for 1 h at a flow rate of 50 mL min^{-1}. All measurements were performed at 0-3°C. The activity was calculated in Wilbur-Anderson Units (WAU) using the following equation 1:

$$WAU = \left(\frac{t_0}{t}\right) - 1 \qquad (1)$$

Where t_0 and t stand for time registered in absence of the enzyme (blank) and in presence of enzyme (tested conditions) respectively.

6. Analysis of metabolomics data with Metaboanalyst 6.0

Metabolomics data were normalized in three steps consisting of row-wise procedures, data transformation and data scaling. First, as row-wise normalization, probabilistic quotient normalization in which each sample is scaled relative to a reference spectrum. The reference consisted of a pooled sample derived from the control group (unexposed control samples) [5]. Row-wise normalization was followed by a data transformation where data were normalized on a logarithmic basis. Finally, data were re-scaled based on pareto scaling, meaning that data were mean-centered and divided by the square root of standard deviation of each variable. One-way analysis of variance (ANOVA) followed by Fisher's LSD post-hoc analysis with $p < 0.05$ was performed to identify significant variations between control, dissolved (Ag+_0.01: 0.01 mg L^{-1} and Ag+_0.02: 0.02 mg L^{-1}) and nanoparticulate (nAg_0.1: 0.1 mg L^{-1} and nAg_0.3: 0.3 mg L^{-1}) silver exposures (**Table S3**). Unsupervised Principal Component Analysis (PCA) and Supervised Partial Least Squares-Discriminant Analysis (PLS-DA) were performed to gain a comprehensive overview of metabolic alterations. Three PLS-DA model was statistically evaluated and validated using cross-validation and permutation testing across different components. The initial two-component model did not achieve statistical significance in permutation testing (p=0.16), indicating limited robustness. Therefore, a third component was included to the model. The three-component model showed good predictive performance (Q^2=0.79) and was statistically significant based on permutation testing (p=0.002, 2/1000), indicating that the observed class separation is unlikely to arise by chance (**Fig. S3**). Important features identified by three-component PLS-DA model were considered as responsive metabolites and were decided based on the variable importance in the projection (VIP) values greater than 1 [6] (**Fig. S3**). Finally, to generate a heatmap, hierarchical clustering was performed on responsive metabolites (ANOVA $p < 0.05$ and VIP > 1) based on Euclidean distance measure and Ward's linkage clustering algorithm. The variations between normalized

concentrations of metabolites, generated by MetaboAnalyst 6.0, were further illustrated by boxplots using OriginPro 2024.

The responsive metabolites were further considered to reveal significantly impacted pathways under exposure to both Ag^{+} and nAg. Pathway analysis was performed with MetaboAnalyst 6.0 based on KEGG pathway built-in metabolic library of green alga *Chlamydomonas reinhardtii* [7]. Pathways exceeding a threshold of 0.1 were considered significantly altered.

Table S1. Synthetic freshwater medium (SFM)+ Si Composition

Compound	Final concentration (mol L^{-1})
HEPES	1.00×10^{-3}
$Ca(NO_3)_2 \times 4H_2O$	2.10×10^{-4}
$MgSO_4 \times 7H_2O$	2.00×10^{-4}
$K_2HPO_4 x 3H_2O$	1.32×10^{-5}
$NaNO_3$	3.50×10^{-4}
Na_2CO_3	1.90×10^{-4}
H_3BO_3	1.60×10^{-5}
$Na_2SiO_3 x 9H_2O$	5.00×10^{-4}
**Vitamins***	
Vitamin B12	1.50×10^{-10}
Biotin	4.10×10^{-9}
Thiamine-HCl	3.00×10^{-7}
Niacinamide	8.00×10^{-10}
**Trace metals solution***	
$Na_2EDTA \times 2 H_2O$	1.17×10^{-5}
$FeCl_3 \times 6 H_2O$	1.17×10^{-5}
K_2CrO_4	9.99×10^{-9}
$CoCl_2 \times 6H_2O$	4.20×10^{-8}
$CuSO_4 \times 5H_2O$	1.00×10^{-8}
$MnCl_2 \times 4H_2O$	9.00×10^{-7}
$Na_2MoO_4 x 2H_2O$	7.81×10^{-8}
$NiSO_4 x 6H_2O$	1.03×10^{-8}
H_2SeO_3	1.01×10^{-8}
Na_3VO_4	1.00×10^{-8}
$ZnSO_4 \times 7H_2O$	7.65×10^{-8}

(*) These compounds were not present in exposure media

Table S2. List of metabolites and the MS parameters for LC-MS targeted metabolomics.

Compound	Retention time (min)	Precursor ion (m/z)	Product ions				
			Quant ion (m/z)	Collision energy (V)	Qual ion (m/z)	Collision energy (V)	Fragmentor (V)
**Amino acids**							
Phenylalanine	2.95	166.1	120.1	13	103	29	80
Leucine	3.38	132.1	86.1	9	30.2	17	75
Tryptophan	3.41	205.1	188	8	146	20	80
Isoleucine	3.75	132.1	86.1	9	44.2	25	75
Methionine	4.22	150.1	104	9	56.1	17	75
Valine	4.95	118.1	72.1	9	55.1	25	70
Proline	4.96	116.1	70.1	17	43.2	37	75
Tyrosine	5.01	182.1	136.1	13	91.1	33	85
Cysteine	5.63	122	59.1	29	76	13	65
Alanine	6.61	90.1	44.2	9	45.3	40	40
Threonine	6.72	120.1	74.1	9	56.1	17	75
Homoserine	6.91	120.1	74.1	9	56.1	21	70
Glycine	7.00	76	30.3	12	-	-	35
Glutamine	7.23	147.1	84.1	17	130.1	9	80
Serine	7.26	106.1	88.1	8	42.2	24	67
Asparagine	7.31	133.1	87.1	5	74	17	75
Glutamic acid	7.68	148.1	84.1	17	130	5	75
Citrulline	7.89	176.1	159.1	9	70.1	25	80
Aspartic acid	8.38	134	88.1	9	74	13	70

Histidine	9.06	156.1	110.1	13	83.1	29	90
Arginine	9.54	175.1	70.1	24	60.1	12	100
Lysine	10.16	147.1	84.1	17	130.1	9	75
Ornithine	10.28	133.1	116	8	70	20	76
**Antioxidants**							
Glutathione reduced	1.22	308.1	179	12	162	16	91
Chlorogenic acid	6.19	353.1	191.1	16	-	-	102
Curcumin	6.33	367.1	217.1	8	149.1	16	112
Vanillic acid	6.60	167	152.1	12	108	20	82
2-hydroxycinnamic acid	7.37	163	119.1	12	117.1	28	81
L-Dehydroascorbic acid	8.00	173	158.1	12	-	-	174
4-(Trifuoromethyl)cinnamic acid	8.26	215	171.1	12	151.1	20	87
a-Tocopherol	11.00	431.4	165.1	24	69.1	40	142
**Organic Acids/Phenolics**							
Glycolic acid	2.04	75	47	8	72.9	8	46
Malic acid	2.07	133	114.9	8	71	16	76
Citric acid	2.17	191	110.8	12	86.9	16	82
Lactic acid	2.23	89.1	43.1	4	-	-	66
Succinic acid	2.31	117	72.9	12	98.9	8	66
Pyruvic acid	2.36	87	43.1	4	-	-	66
Gallic acid	2.49	169	125.1	12	79	24	92
Glutaric acid	2.62	131	86.9	12	112.9	8	71
Fumaric acid	2.67	115	70.9	4	-	-	56
Ascorbic acid	2.67	175	114.9	12	-	-	87
Caffeic acid	4.58	179	135.1	16	-	-	94

p-coumaric acid	4.87	163	119.1	16	93.1	36	87
Ferulic acid	5.09	193.1	134.1	16	178.1	12	87
Benzoic acid	5.21	121	77.1	12	-	-	77
Salicyllic acid	5.96	137	93	20	65.1	36	82
**Sugar and Sugar Alcohol**							
Ribose	1.18	149	89	4	-	-	76
L-fucose	1.35	163.1	89	0	59.1	12	76
Arabinose	1.43	149	89	4	-	-	76
Xylose	1.43	149	89	4	-	-	76
Ribitol	1.61	151.1	89	8	71.1	16	97
Xylitol	1.61	151.1	89	12	-	-	97
Fructose	1.72	179.1	89	4	-	-	71
Mannose	1.93	179.1	89	16	-	-	71
Galactose	2.19	179.1	89	16	-	-	71
Glucose	2.19	179.1	89	16	-	-	71
Sucrose	3.81	341.1	179	20	-	-	148
Maltose	4.26	341.1	161.1	4	-	-	123
Lactose	4.57	341.1	161.1	4	-	-	123
Trehalose	4.79	341.1	179	12	-	-	154
Raffinose	6.03	503.2	179	20	221	32	174
Galactinol	6.17	341.1	179	12	-	-	133
**Fatty Acids**							
Linolenic acid	4.33	323.2	277.1	4	45.1	40	87
Myristic acid	4.64	273.2	227.2	4	45.1	8	56
Linoleic acid	4.91	325.2	279.1	4	45.1	28	87
Pentadecanoic acid	5.17	287.2	241.2	4	45.1	16	71

Palmitic acid	5.70	301.2	255.2	4	45.1	20	36
Heptadecanoic acid	6.14	315.3	269.2	4	45.2	28	76
Stearic acid	6.49	329.3	283.2	4	45.1	32	72
Arachidic acid	7.05	357.3	311.3	4	45.1	32	82
**Nucleobase/side/tide**							
Cytosine	1.94	112.1	95	20	40.1	20	84
CMP	2.76	324.1	112	16	95	40	84
Cytidine	2.90	244.1	112	12	95	40	84
Adenine	3.08	136.1	119	24	92	32	84
Guanine	3.34	152.1	135	20	110	24	84
uracil	3.52	113	70	10	96	20	84
AMP	4.84	348.1	136	20	97	32	84
Hypoxanthine	5.28	137	110	24	55.1	36	148
Uridine	6.33	245.1	113	8	70	40	84
Xanthine	6.40	153	110	20	55.1	36	84
Adenosine	6.67	268.1	136	20	119	40	84
Thymine	6.71	127.1	110	16	54.1	28	84
Guanosine	6.91	284.1	152	12	135	40	84
Inosine	6.91	269.1	137	16	110	40	84
Thymidine	7.28	243.1	127	8	117	8	84
**Vitamins**							
Thiamine	1.64	266.1	122	16	81	36	77
Pyridoxamine	1.70	169.1	152	12	134	24	72
Pyridoxal	2.04	168.1	150	12	94	24	72
Pyridoxine	2.09	170.1	152	12	134	24	82
Nicotinamide riboside	2.11	256.1	124	8	123.1	8	72

Nicotinic acid	2.45	124	80	24	78	24	92
Nicotinamide	2.41	123.1	80	24	53.1	32	92
Pantothenic acid	3.37	220.1	202	12	90	12	82
Cobalamin	3.45	678.7	359	20	147	40	134
Folic acid	3.54	442.2	294.9	16	176	40	72
Riboflavin	3.79	377.2	243	24	172	40	149
Biotin	3.77	245.1	227	12	92	36	77
**Additional metabolites**							
Beta-carotene	10.93	537.5	177	16	95	36	154
Fucoxanthin	3.29	659.4	641.3	12	109	36	154
Lutein	5.75	569.4	145	40	119.1	40	149
Lycopene	10.92	537.5	81	28	69	36	164
Putrescine	2.59	89.1	72	8	30.2	28	66
Spermidine	1.48	146.2	72	16	30.1	40	87
Spermine	1.41	203.2	129.1	12	112	20	92

Table S3. Key features identified by One-way ANOVA and Fisher's post-hoc analysis in *C. meneghiniana* exposed to two concentrations of Ag^+ (Ag^+; 0.01 and 0.02 mg L^{-1}) and two concentrations of nAg (nAg; 0.1 and 0.3 mg L^{-1}). Data were normalized using probabilistic quotient normalization (PQN) with pooled control sample as reference, followed by logarithmic based normalization and pareto-based scaling.

Metabolites	f.value	p.value	-log10(p)	FDR	Fisher's LSD
**Spermidine**	51.431	1.23E-06	5.9084	4.78E-05	Ag+_0.02 - CTR; nAg_0.1 - CTR; nAg_0.3 - CTR; Ag+_0.02 - Ag+_0.01; nAg_0.1 - Ag+_0.01; nAg_0.3 - Ag+_0.01; Ag+_0.02 - nAg_0.1; nAg_0.3 - nAg_0.1
**Inosine**	48.73	1.59E-06	5.7978	4.78E-05	CTR - Ag+_0.01; nAg_0.1 - CTR; nAg_0.3 - CTR; Ag+_0.02 - Ag+_0.01; nAg_0.1 - Ag+_0.01; nAg_0.3 - Ag+_0.01; nAg_0.1 - Ag+_0.02; nAg_0.3 - Ag+_0.02; nAg_0.3 - nAg_0.1
**Uridine**	34.626	7.84E-06	5.1056	0.00015681	Ag+_0.02 - CTR; nAg_0.1 - CTR; nAg_0.3 - CTR; Ag+_0.02 - Ag+_0.01; nAg_0.1 - Ag+_0.01; nAg_0.3 - Ag+_0.01; nAg_0.3 - Ag+_0.02; nAg_0.3 - nAg_0.1
**Citric acid**	24.806	3.57E-05	4.4479	0.00053476	Ag+_0.01 - CTR; Ag+_0.02 - CTR; nAg_0.1 - CTR; nAg_0.3 - CTR; Ag+_0.02 - Ag+_0.01; nAg_0.3 - Ag+_0.01; Ag+_0.02 - nAg_0.1; nAg_0.3 - nAg_0.1
**Glutamine**	22.46	5.54E-05	4.2562	0.00066521	CTR - nAg_0.1; CTR - nAg_0.3; Ag+_0.01 - nAg_0.1; Ag+_0.01 - nAg_0.3; Ag+_0.02 - nAg_0.1; Ag+_0.02 - nAg_0.3; nAg_0.1 - nAg_0.3
**Tyrosine**	21.177	7.18E-05	4.1438	0.00067009	Ag+_0.01 - CTR; Ag+_0.02 - CTR; nAg_0.1 - CTR; nAg_0.3 - CTR; Ag+_0.02 - Ag+_0.01; nAg_0.1 - Ag+_0.01; nAg_0.3 - Ag+_0.01
**Aspartic acid**	20.771	7.82E-05	4.1069	0.00067009	CTR - nAg_0.1; CTR - nAg_0.3; Ag+_0.01 - nAg_0.1; Ag+_0.01 - nAg_0.3; Ag+_0.02 - nAg_0.1; Ag+_0.02 - nAg_0.3; nAg_0.1 - nAg_0.3
**Guanosine**	17.633	0.00015882	3.7991	0.0011911	nAg_0.1 - CTR; nAg_0.3 - CTR; nAg_0.1 - Ag+_0.01; nAg_0.3 - Ag+_0.01; nAg_0.3 - Ag+_0.02; nAg_0.3 - nAg_0.1
**Malic acid**	16.277	0.00022317	3.6514	0.0014878	CTR - nAg_0.3; Ag+_0.01 - nAg_0.3; Ag+_0.02 - nAg_0.3; nAg_0.1 - nAg_0.3
**Leucine**	12.948	0.00057625	3.2394	0.0034575	CTR - nAg_0.3; Ag+_0.01 - nAg_0.1; Ag+_0.01 - nAg_0.3; Ag+_0.02 - nAg_0.3; nAg_0.1 - nAg_0.3
**Alanine**	10.206	0.0014794	2.8299	0.0080693	nAg_0.1 - CTR; nAg_0.3 - CTR; nAg_0.3 - Ag+_0.01; nAg_0.3 - Ag+_0.02; nAg_0.3 - nAg_0.1
**Spermine**	9.1716	0.0022224	2.6532	0.011112	CTR - Ag+_0.02; CTR - nAg_0.3; Ag+_0.01 - Ag+_0.02; Ag+_0.01 - nAg_0.3; nAg_0.1 - Ag+_0.02; nAg_0.1 - nAg_0.3
**Methionine**	7.9668	0.0037359	2.4276	0.017243	Ag+_0.02 - CTR; nAg_0.1 - CTR; nAg_0.3 - CTR; Ag+_0.02 - Ag+_0.01; nAg_0.1 - Ag+_0.01; nAg_0.3 - Ag+_0.01
**Fumaric acid**	7.4818	0.0046789	2.3299	0.020052	CTR - nAg_0.3; Ag+_0.01 - nAg_0.3; Ag+_0.02 - nAg_0.3; nAg_0.1 - nAg_0.3
**Tryptophan**	6.9405	0.0060896	2.2154	0.024358	Ag+_0.01 - CTR; Ag+_0.02 - CTR; nAg_0.1 - CTR; nAg_0.3 - CTR
**Cytidine**	6.7691	0.0066389	2.1779	0.024896	nAg_0.3 - CTR; nAg_0.1 - Ag+_0.01; nAg_0.3 - Ag+_0.01; nAg_0.3 - Ag+_0.02; nAg_0.3 - nAg_0.1

**AMP**	6.3504	0.0082503	2.0835	0.029119	nAg_0.3 - CTR; nAg_0.1 - Ag+_0.01; nAg_0.3 - Ag+_0.01; nAg_0.3 - Ag+_0.02; nAg_0.3 - nAg_0.1
**Ornithine**	5.9716	0.010126	1.9946	0.033118	CTR - Ag+_0.01; CTR - Ag+_0.02; CTR - nAg_0.3; nAg_0.1 - Ag+_0.02
**Glutathione reduced**	5.9083	0.010487	1.9793	0.033118	CTR - Ag+_0.02; CTR - nAg_0.1; CTR - nAg_0.3; Ag+_0.01 - Ag+_0.02
**Arginine**	5.6955	0.011819	1.9274	0.035458	CTR - Ag+_0.02; Ag+_0.01 - Ag+_0.02; nAg_0.1 - Ag+_0.02; nAg_0.3 - Ag+_0.02
**Citrulline**	5.5548	0.012812	1.8924	0.036606	CTR - Ag+_0.02; Ag+_0.01 - Ag+_0.02; nAg_0.1 - Ag+_0.02; nAg_0.3 - Ag+_0.02
**Glycine**	5.3097	0.014788	1.8301	0.039744	Ag+_0.01 - CTR; nAg_0.3 - CTR; Ag+_0.01 - Ag+_0.02; Ag+_0.01 - nAg_0.1; nAg_0.3 - Ag+_0.02
**Guanine**	5.2597	0.015235	1.8172	0.039744	CTR - Ag+_0.02; Ag+_0.01 - Ag+_0.02; nAg_0.1 - Ag+_0.02
**Proline**	5.0458	0.017339	1.761	0.043347	Ag+_0.01 - CTR; Ag+_0.01 - nAg_0.3; Ag+_0.02 - nAg_0.3; nAg_0.1 - nAg_0.3
**Succinic acid**	4.9057	0.018904	1.7234	0.045369	Ag+_0.02 - CTR; nAg_0.1 - CTR; nAg_0.3 - CTR; nAg_0.1 - Ag+_0.01; nAg_0.3 - Ag+_0.01

Table S4. Key features identified by One-way ANOVA and Fisher's post-hoc analysis in *C. meneghiniana* exposed to Ag^+ (Ag^+; 0.01 mg L^{-1}) and nAg (nAg; 0.3 mg L^{-1}). Data were normalized using probabilistic quotient normalization (PQN) with pooled control sample as reference, followed by logarithmic based normalization and pareto-based scaling.

	f.value	p.value	-log10(p)	FDR	Fisher's LSD
**Aspartic acid**	64.50	8.8E-05	4.06	0.00	Ag+_0.01 - CTR; CTR - nAg_0.3; Ag+_0.01 - nAg_0.3
**Inosine**	62.51	9.6E-05	4.02	0.00	CTR – Ag+_0.01; nAg_0.3 - CTR; nAg_0.3 – Ag+_0.01
**Spermidine**	59.18	1.1E-04	3.95	0.00	nAg_0.3 - CTR; nAg_0.3 – Ag+_0.01
**Uridine**	59.17	1.1E-04	3.95	0.00	nAg_0.3 - CTR; nAg_0.3 – Ag+_0.01
**Glutamine**	48.66	2.0E-04	3.71	0.00	CTR - nAg_0.3; Ag+_0.01 - nAg_0.3
**Leucine**	40.90	3.2E-04	3.50	0.00	CTR - nAg_0.3; Ag+_0.01 - nAg_0.3
**Tyrosine**	38.26	3.8E-04	3.42	0.00	Ag+_0.01 - CTR; nAg_0.3 - CTR; nAg_0.3 – Ag+_0.01
**Malic acid**	34.68	5.0E-04	3.30	0.00	CTR - nAg_0.3; Ag+_0.01 - nAg_0.3
**Citric acid**	29.86	7.6E-04	3.12	0.01	nAg_0.3 - CTR; nAg_0.3 – Ag+_0.01
**Alanine**	24.46	1.3E-03	2.88	0.01	nAg_0.3 - CTR; nAg_0.3 – Ag+_0.01
**Guanosine**	22.72	1.6E-03	2.80	0.01	nAg_0.3 - CTR; nAg_0.3 – Ag+_0.01
**Spermine**	14.07	5.4E-03	2.27	0.03	CTR - nAg_0.3; Ag+_0.01 - nAg_0.3
**Fumaric acid**	13.38	6.1E-03	2.21	0.03	CTR - nAg_0.3; Ag+_0.01 - nAg_0.3
**AMP**	12.56	7.2E-03	2.14	0.03	nAg_0.3 - CTR; nAg_0.3 – Ag+_0.01
**Proline**	11.74	8.4E-03	2.07	0.03	Ag+_0.01 - CTR; Ag+_0.01 - nAg_0.3
**Homoserine**	11.43	9.0E-03	2.05	0.03	Ag+_0.01 - CTR; nAg_0.3 - CTR
**Tryptophan**	9.96	1.2E-02	1.91	0.04	Ag+_0.01 - CTR; nAg_0.3 - CTR

Table S5. Detailed results from the pathway analysis. Statistical p values obtained from enrichment analysis were adjusted for multiple testings. **Total** stands for total number of compounds in the pathway; **Hits** represent the matched number from user uploaded data; **Raw p** correspond to original p value calculated from the enrichment analysis; **Holm p** is the p value adjusted by Holm-Bonferroni method; **FDR p** stands for p value adjusted using False Discovery Rate; and the **Impact** correspond ton impact value calculated from pathway topology analysis.

	**Total**	**Expected**	**Hits**	**Raw p**	**-LOG10(p)**	**Holm adjust**	**FDR**	**Impact**
**Arginine biosynthesis**	18	0.508	6	4.40E-06	5.3561	0.00034356	0.00034356	0.38926
**Alanine, aspartate and glutamate metabolism**	19	0.53622	4	0.0014914	2.8264	0.11484	0.04752	0.44872
**Citrate cycle (TCA cycle)**	20	0.56444	4	0.0018277	2.7381	0.1389	0.04752	0.19704
**Glyoxylate and dicarboxylate metabolism**	25	0.70555	4	0.0043222	2.3643	0.32416	0.084282	0.32238
**Glutathione metabolism**	27	0.76199	4	0.0057637	2.2393	0.42651	0.089913	0.42418
**Glycine, serine and threonine metabolism**	30	0.84666	4	0.0084817	2.0715	0.61916	0.11026	0.18948
**Carbon fixation by Calvin cycle**	21	0.59266	3	0.019149	1.7179	1	0.21337	0.05923
**Lysine biosynthesis**	9	0.254	2	0.024516	1.6105	1	0.23904	0
**Arginine and proline metabolism**	25	0.70555	3	0.030686	1.5131	1	0.26594	0.29282
**Galactose metabolism**	27	0.76199	3	0.037569	1.4252	1	0.29304	0.26115
**Neomycin, kanamycin and gentamicin biosynthesis**	2	0.056444	1	0.055673	1.2544	1	0.39477	0
**Linoleic acid metabolism**	3	0.084666	1	0.082374	1.0842	1	0.44501	1
**Pyrimidine metabolism**	39	1.1007	3	0.093571	1.0289	1	0.44501	0.10017
**Cysteine and methionine metabolism**	41	1.1571	3	0.10512	0.97831	1	0.44501	0.17595
**One carbon pool by folate**	20	0.56444	2	0.10685	0.97122	1	0.44501	0.12376
**Starch and sucrose metabolism**	20	0.56444	2	0.10685	0.97122	1	0.44501	0.15385
**Cyanoamino acid metabolism**	4	0.11289	1	0.10834	0.96519	1	0.44501	0
**Biosynthesis of various plant secondary metabolites**	4	0.11289	1	0.10834	0.96519	1	0.44501	0
**Purine metabolism**	69	1.9473	4	0.12431	0.90548	1	0.44501	0.16929
**Phenylalanine, tyrosine and tryptophan biosynthesis**	22	0.62088	2	0.12552	0.9013	1	0.44501	0.02002
**Pyruvate metabolism**	22	0.62088	2	0.12552	0.9013	1	0.44501	0.1311

**Tyrosine metabolism**	22	0.62088	2	0.12552	0.9013	1	0.44501	0.22857
**Isoquinoline alkaloid biosynthesis**	6	0.16933	1	0.15817	0.80087	1	0.53641	0.5
**Monobactam biosynthesis**	8	0.22578	1	0.2053	0.68761	1	0.66723	0
**Nitrogen metabolism**	11	0.31044	1	0.27125	0.56664	1	0.84629	0
**Vitamin B6 metabolism**	12	0.33866	1	0.29203	0.53457	1	0.8698	0
**Fructose and mannose metabolism**	13	0.36689	1	0.31224	0.50552	1	0.8698	0
**Nicotinate and nicotinamide metabolism**	13	0.36689	1	0.31224	0.50552	1	0.8698	0
**Selenocompound metabolism**	16	0.45155	1	0.36957	0.4323	1	0.96089	0
**Propanoate metabolism**	16	0.45155	1	0.36957	0.4323	1	0.96089	0
**Thiamine metabolism**	20	0.56444	1	0.43888	0.35766	1	1	0
**Valine, leucine and isoleucine biosynthesis**	22	0.62088	1	0.47071	0.32725	1	1	0
**Glucosinolate biosynthesis**	22	0.62088	1	0.47071	0.32725	1	1	0
**Lipoic acid metabolism**	24	0.67733	1	0.50079	0.30035	1	1	0.0016
**Tryptophan metabolism**	27	0.76199	1	0.54283	0.26533	1	1	0.16364
**Valine, leucine and isoleucine degradation**	32	0.9031	1	0.60542	0.21795	1	1	0
**Amino sugar and nucleotide sugar metabolism**	37	1.0442	1	0.65968	0.18067	1	1	0
**Ubiquinone and other terpenoid-quinone biosynthesis**	41	1.1571	1	0.69782	0.15625	1	1	0

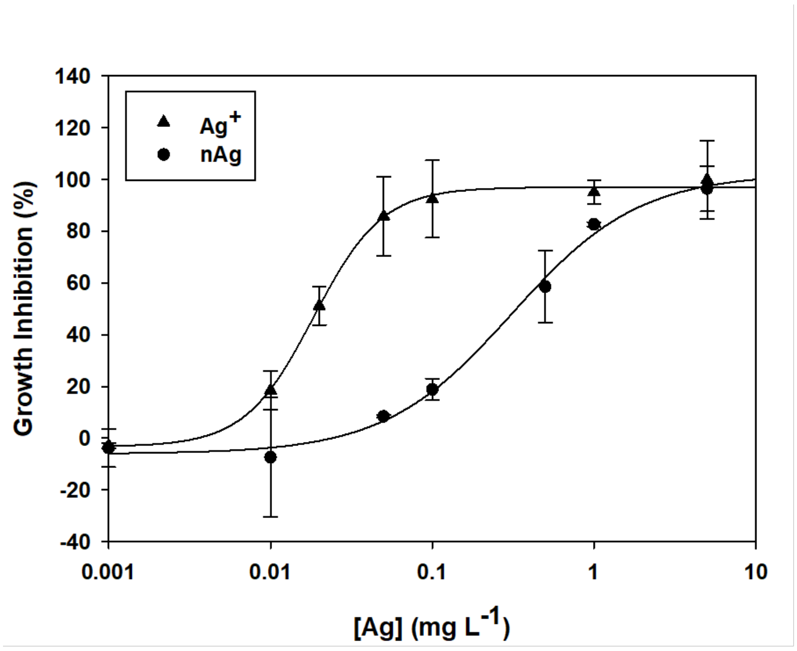

Figure S1. *C. meneghiniana* growth inhibition curve after 72-h exposure to different concentrations of Ag^+ and nAg.

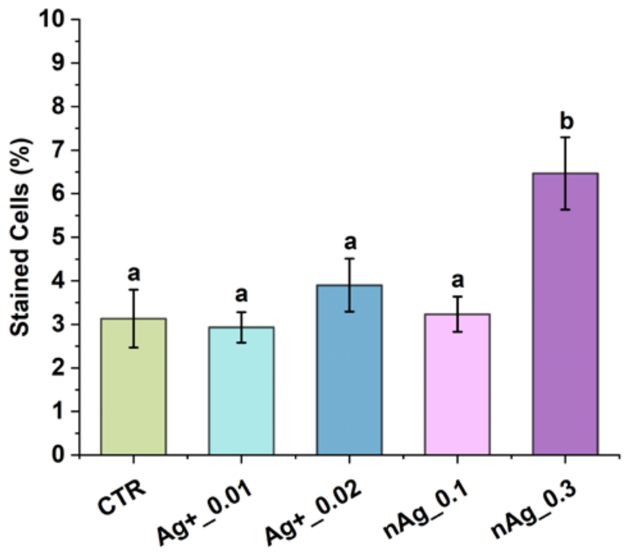

Figure S2. Percentage of PI-stained *C. meneghiniana* cells after 2-h exposure to two concentrations dissolved silver (Ag^+; 0.01 and 0.02 mg L^{-1}) and nanoparticulate silver (nAg; 0.1 and 0.3 mg L^{-1}). Error bars denote standard deviation (n=3).

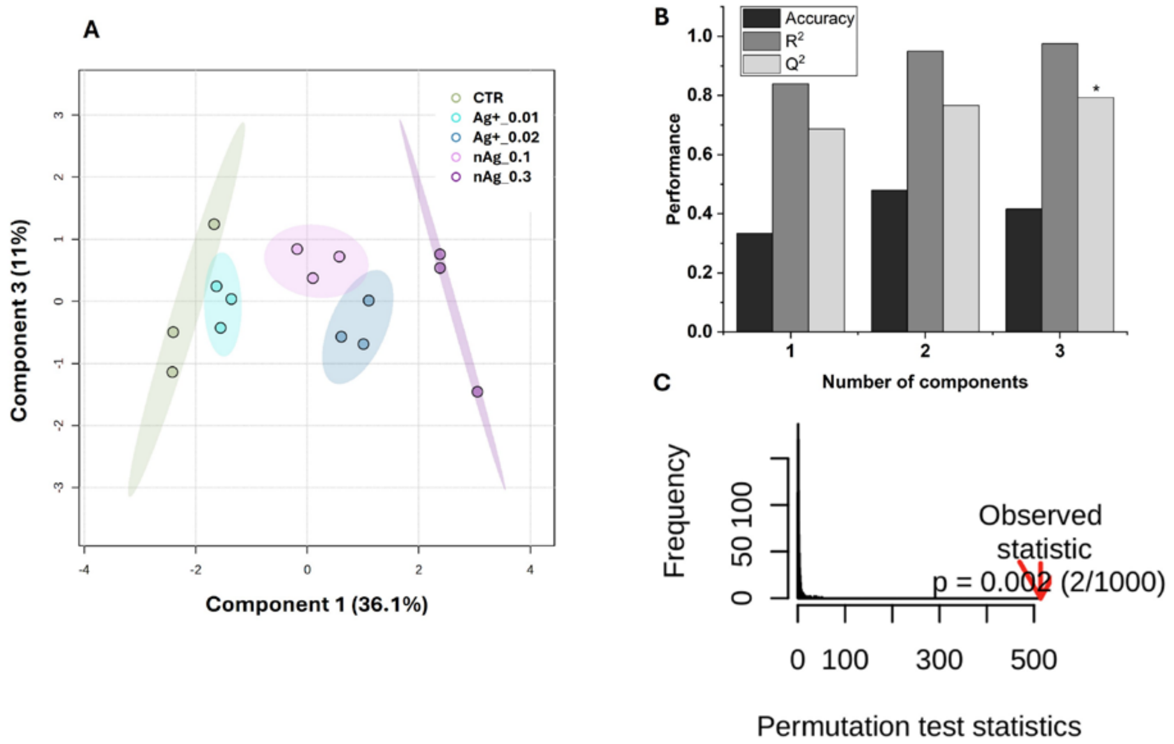

Figure S3. Three-component validated partial least squares discriminant analysis (PLS-DA) model assessed by cross validation and permutation testing. (A) Score plot showing sample distribution based on first three components. (B) Classification performance of PLS-DA models using varying numbers of components. The asterisk indicates the best classifier. (C) PLS-DA model validation based on separation distance. The p value based on permutation is p=0.002 (2/1000).

The extended model including a third component improved discrimination between Ag^+_0.02 and nAg_0.1 treatment conditions (Fig. S#A). Cross validation showed a strong and consistent increase in predictive performance, with Q^2 reaching 0.79 (Fig. S#B). Model fit was high, with R^2 values up to 0.97. Importantly, the relatively small difference between R^2 and Q^2 (0.18) indicates that the model is not overfitted. Although classification accuracy was moderate and showed some variability, this is expected in multi-class models with limited sample sizes and does not detract from the model's predictive validity. Finally, permutation testing (n=1000) yielded an empirical p-value of 0.002, demonstrating strong statistical significance and confirming that the observed class separation is unlikely to arise by chance (Fig. S#C).

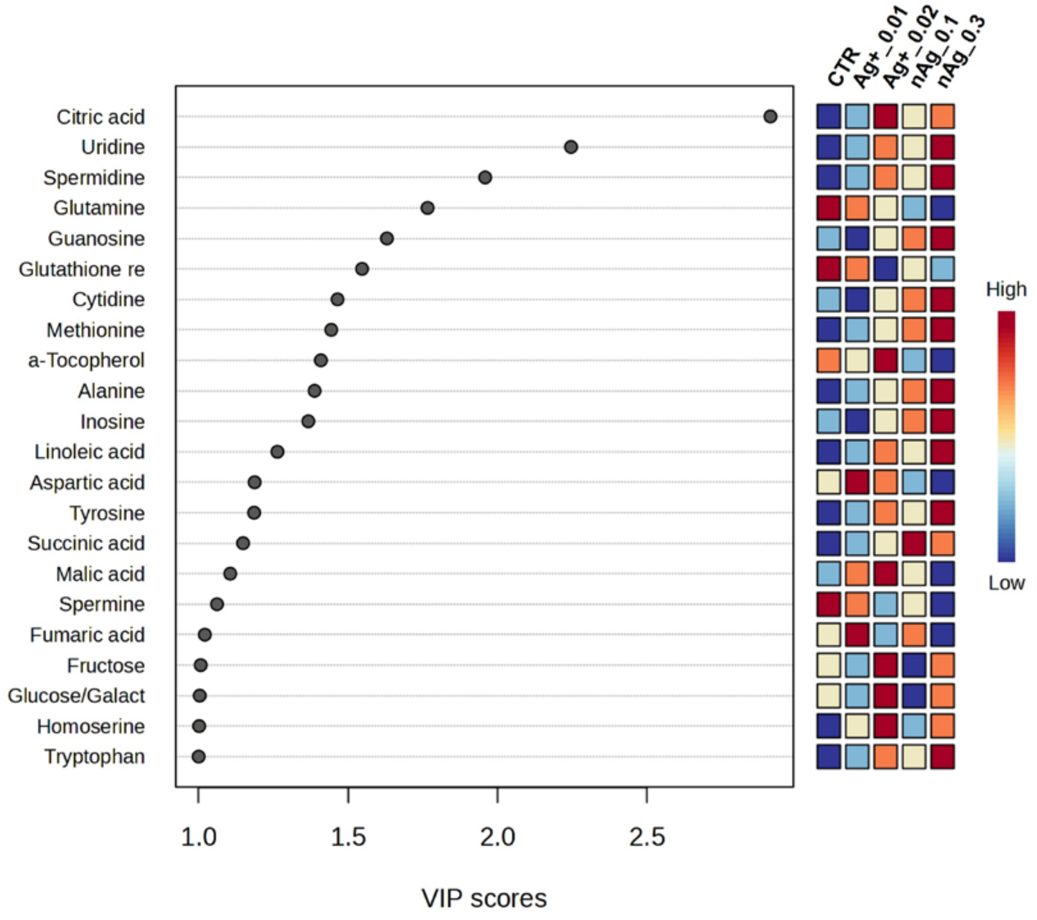

Figure S4. Variable Importance in Projection (VIP) scores from three-component PLS-DA model, discriminating between the control (CTR), two concentrations of dissolved silver (Ag+_0.01: 0.01 mg L^{-1} Ag and Ag+_0.02: 0.02 mg L^{-1} Ag), and two concentrations of nanoparticulate silver (nAg_0.1: 0.1 mg L^{-1} Ag and nAg_0.3: 0.3 mg L^{-1} Ag). Colored boxes on the right indicate the relative abundance of each metabolite across treatment groups. Only metabolites with a VIP score > 1 were considered significant and are displayed.

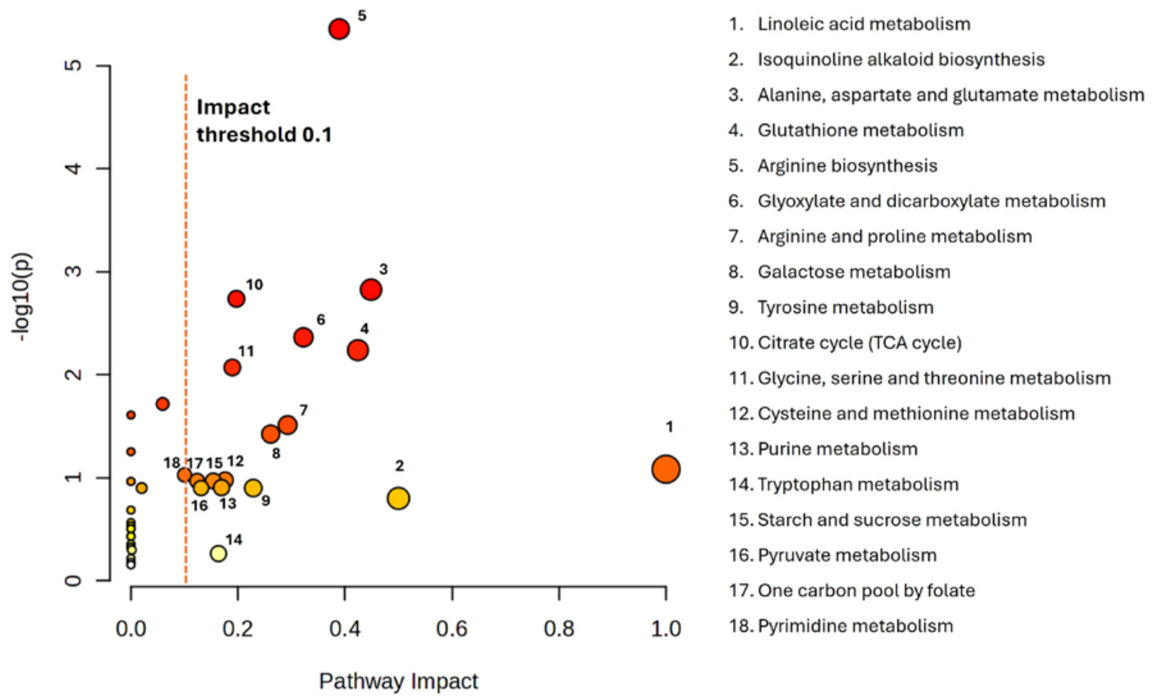

Figure S5. Results of pathway analysis based on 30 responsive metabolites, determined by ANOVA (<0.05) and variable importance in projection (VIP>1), in diatom *C. meneghiniana* exposed to two concentrations of dissolved silver (Ag^+; 0.01 and 0.02 mg L^{-1}) and nanoparticulate silver (nAg; 0.1 and 0.3 mg L^{-1}). The node color changes based on p-value, from lowest to highest p-value, from red to yellow respectively. The node size stands for pathway impact value, where bigger sized nodes representing higher impact. Detailed results from the pathway analysis could be found in **Table S5**.

To assess the impact of both Ag forms on *C. meneghiniana* metabolism and to identify altered biochemical pathways, we performed a pathway analysis considering the determined responsive metabolites for both Ag^+ and nAg treatments. Pathway analysis revealed eighteen significantly altered biochemical pathways (threshold < 0.1). These included linoleic acid metabolism, isoquinoline alkaloid biosynthesis, alanine, aspartate and glutamate metabolism, glutathione metabolism, arginine biosynthesis, glyoxylate and dicarboxylate metabolism, arginine and proline metabolism, galactose metabolism, tyrosine metabolism, citrate cycle

(TCA cycle), glycine, serine and threonine metabolism, cysteine and methionine metabolism, purine metabolism, tryptophan metabolism, starch and sucrose metabolism, pyruvate metabolism, one carbon pool by folate metabolism and pyrimidine metabolism.

Exposure to high concentrations of nAg (1 mg L^{-1}) and Ag^+ (40.7 µg L^{-1}) altered similar metabolic pathways in *P. malhamensis* after 2 hours[8]. On the other hand, low concentrations of nAg (0.09 and 0.2 mg L^{-1}) impacted comparable metabolic pathways in *C. vulgaris* after long exposure time (7 days) including arginine and proline metabolism, glutathione metabolism, glyoxylate and dicarboxylate metabolism, alanine aspartate and glutamate metabolism and glycine, serine and threonine metabolism [9]. Moreover, the impact of low concentrations of nAg (0.01, 0.1 and 1 mg L^{-1}) and Ag^+ (0.1, 1 and 10 µg L^{-1}) on cyanobacteria *N. sphaeroides* was studied after 96 hours of exposure where similar metabolic pathways were altered [10].

This analysis highlights that even short-term (2 h) exposure to low concentrations of nAg and Ag^+ led to broad metabolic reprogramming in *C. meneghiniana*, underscoring their potential as sensitive biomarkers for early nanoparticle toxicity detection.

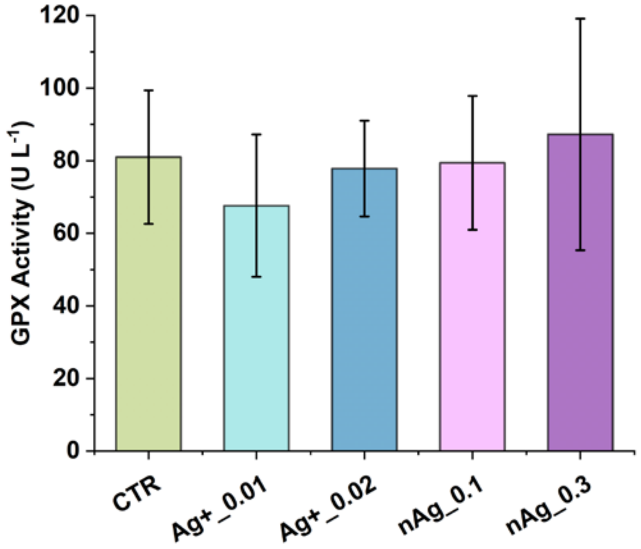

Figure S6. Glutathione peroxidase activity in *C. meneghiniana* after 2-h exposure to two concentrations of dissolved silver (Ag^{+}; 0.01 and 0.02 mg L^{-1}) and nanoparticulate silver (nAg; 0.1 and 0.3 mg L^{-1}). Error bars denote standard deviation (n=4).

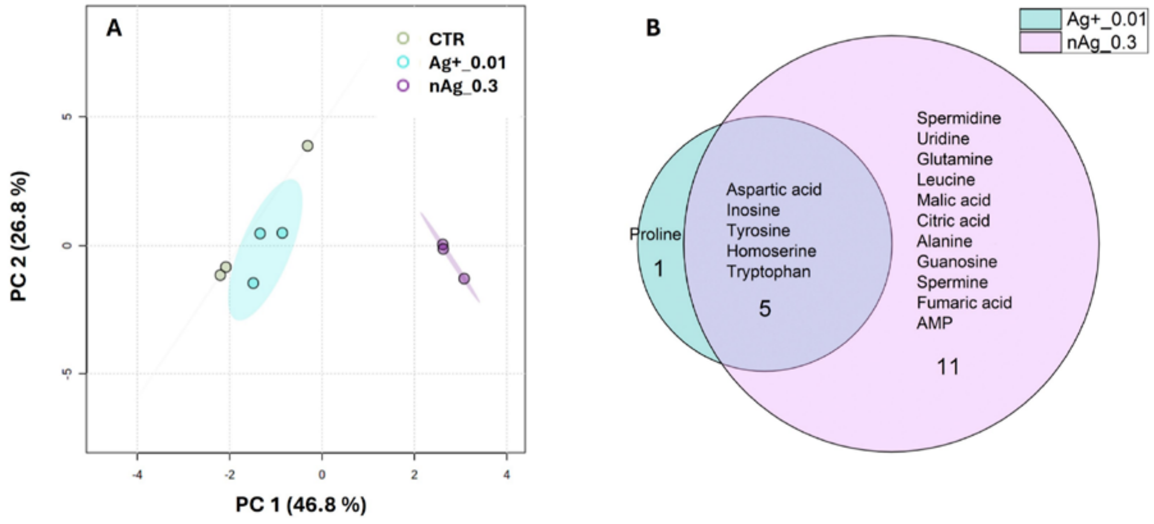

Figure S7. Comparative metabolomic responses of *C. meneghiniana* exposed to nanoparticulate and equivalent dissolved silver concentration. (A) Principal component analysis (PCA) showing separation among control (CTR), silver nanoparticle (nAg_0.3: 0.3 mg L^{-1}) and dissolved silver (Ag^{+}_0.01: 0.01 mg L^{-1}) and (B) Venn diagram illustrating silver form–specific and shared responsive metabolites in *C. meneghiniana* after 2-h exposure to nAg (0.3 mg L^{-1}) and the corresponding dissolved silver concentration (Ag^{+}_0.01: 0.01 mg L^{-1}). Differential metabolites were identified based on ANOVA followed by Fisher's LSD post hoc test.

References

1. Kantarciyan A, Segovia-Campos I, Slaveykova VI. Evaluating cell surface extraction methods for improved assessment of silver nanoparticle bioaccumulation. Aquat Toxicol. 2025;283:107340. Epub 20250327. doi: 10.1016/j.aquatox.2025.107340. PubMed PMID: 40203782.

2. Lackmann C, Velki M, Bjedov D, Ečimović S, Seiler T-B, Hollert H. Commercial preparations of pesticides exert higher toxicity and cause changes at subcellular level in earthworm Eisenia andrei. Environmental Sciences Europe. 2021;33(1). doi: 10.1186/s12302-021-00455-5.

3. Wilbur KM, Anderson NG. Electrometric and Colorimetric Determination of Carbonic Anhydrase. Journal of Biological Chemistry. 1948;176(1):147-54. doi: 10.1016/s0021-9258(18)51011-5.

4. Fernández PA, Roleda MY, Rautenberger R, Hurd CL. Carbonic anhydrase activity in seaweeds: overview and recommendations for measuring activity with an electrometric method, using Macrocystis pyrifera as a model species. Marine Biology. 2018;165(5). doi: 10.1007/s00227-018-3348-5.

5. Dieterle F, Ross A, Schlotterbeck G, Senn H. Probabilistic quotient normalization as robust method to account for dilution of complex biological mixtures. Application in 1H NMR metabonomics. Anal Chem. 2006;78(13):4281-90. doi: 10.1021/ac051632c. PubMed PMID: 16808434.

6. Jung Y, Ahn YG, Kim HK, Moon BC, Lee AY, Ryu DH, et al. Characterization of dandelion species using 1H NMR- and GC-MS-based metabolite profiling. Analyst. 2011;136(20):4222-31. Epub 20110826. doi: 10.1039/c1an15403f. PubMed PMID: 21874166.

7. McNabney DWG, Mangal V, Kirkwood AE, Simmons DDB. Phytoplankton metabolite profiles from two Lake Ontario Areas of Concern reveal differences associated with taxonomic

community composition. Sci Total Environ. 2023;871:162042. Epub 20230206. doi: 10.1016/j.scitotenv.2023.162042. PubMed PMID: 36754333.

8. Liu W, Majumdar S, Li W, Keller AA, Slaveykova VI. Metabolomics for early detection of stress in freshwater alga Poterioochromonas malhamensis exposed to silver nanoparticles. Sci Rep. 2020;10(1):20563. Epub 20201125. doi: 10.1038/s41598-020-77521-0. PubMed PMID: 33239722; PubMed Central PMCID: PMCPMC7689461.

9. Qu R, Xie Q, Tian J, Zhou M, Ge F. Metabolomics reveals the inhibition on phosphorus assimilation in Chlorella vulgaris F1068 exposed to AgNPs. Sci Total Environ. 2021;770:145362. Epub 20210123. doi: 10.1016/j.scitotenv.2021.145362. PubMed PMID: 33736381.

10. Huang M, Keller AA, Wang X, Tian L, Wu B, Ji R, et al. Low Concentrations of Silver Nanoparticles and Silver Ions Perturb the Antioxidant Defense System and Nitrogen Metabolism in N(2)-Fixing Cyanobacteria. Environ Sci Technol. 2020;54(24):15996-6005. Epub 20201124. doi: 10.1021/acs.est.0c05300. PubMed PMID: 33232140.
